# Assorted functionality-appended UiO-66-NH_2_ for highly efficient uranium(vi) sorption at acidic/neutral/basic pH†

**DOI:** 10.1039/d0ra00410c

**Published:** 2020-04-09

**Authors:** Sarita Tripathi, B. Sreenivasulu, A. Suresh, C. V. S. Brahmmananda Rao, N. Sivaraman

**Affiliations:** Fuel Chemistry Division, Indira Gandhi Centre for Atomic Research Kalpakkam Tamil Nadu-603102 India sarita17aug@gmail.com sivaram@igcar.gov.in; Homi Bhabha National Institute (HBNI) India

## Abstract

A series of functionalized metal organic frameworks (MOFs) were synthesized by the post-synthetic modification (PSM) of Zr(iv)-containing UiO-66-NH_2_ MOFs using covalent grafting with various functional groups utilizing pendant –NH_2_ moieties. The tethering of amide (with/without pendant carboxylic acid), iminopyridine, phoshinic amide and sulphur-containing functionalities produced a library of eight different UiO-66-NH_2_ derivatives. The functionalized MOFs were characterized by FT-IR spectroscopy, NMR, PXRD, TGA, SEM-EDX and BET surface area analysis. Uranyl ion extraction with the functionalized MOFs was investigated in acidic/neutral/basic conditions (pH 1 to 9). This work presents a comprehensive study of different functionalized MOFs to investigate the effects of various analytical parameters, including pH, contact time, and desorption process. The MOFs as solid phase extractants (SPEs) provide a direct comparison of the sorption efficiencies of different functional groups on a common solid support. A phosphorous-functionalized material, UiO-66-PO-Ph, with enhanced thermal stability (∼500 °C) exhibits the best sorption capacity (∼96%) in an acidic medium (pH 3). The parent MOF UiO-66-NH_2_ (92%) and iminopyridine-functionalized UiO-66-IMP (90%) showed excellent sorption in neutral conditions (pH 7). Amide-containing MOFs UiO-66-AM1 (40%), UiO-66-AMMal (31%) and UiO-66-AMGlu (70%), sulfur-based MOFs UiO-66-SMA (65%) and UiO-66-SSA (27%), and phosphorous-functionalized UiO-66-PO-OPh (50%) displayed maximum sorption in basic conditions (pH 8). The kinetics studies revealed rapid uranium sorption in about 2 h due to the effective binding of uranyl ions with the anchored functional groups of MOFs; quantitative elution of uranyl ions from the MOF framework was carried out with 0.1/0.01 M HNO_3_. The MOFs also exhibit moderate recyclability for uranium sorption and can be regenerated by an acidic solution. The functionalized MOFs alter the stability in acidic/basic media; thus, UiO-66-NH_2_ is a versatile MOF material employed as an SPE for the extraction of radionuclides from aqueous media. This work also provides a platform for the development of new functionalized MOF materials for the efficient sorption of uranium as well as moderate recyclability for its removal, and the potential applications include the removal of uranium from aqueous waste streams.

## Abstract 2

Eight assorted functionalities were anchored on UiO-66-NH_2_*via* PSM strategy displaying MOFs with similar framework but variable uranyl binding affinities. The excellent sorption capacity of UiO-66-PO-Ph makes it efficient uranium sorbent material.

## Graphical abstract

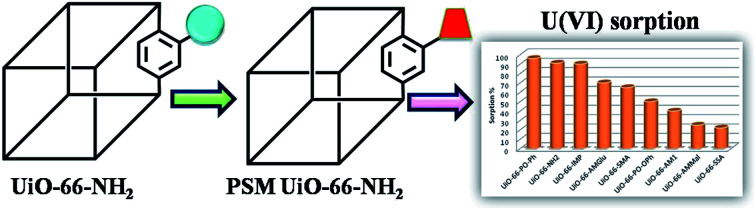

## 1 Introduction

Due to their extensive options in terms of structure design and pore size tunability, metal organic frameworks (MOFs) have prospered enormously in a wide range of applications, such as gas and liquid sorption/separation, catalysis, sensing, drug delivery, magnetism and ion/electrical conductivity.^1–14^ In general, MOFs containing aromatic rings in their backbones provide excellent chemical stability and can be further enhanced by the incorporation of high-valent group (IV) metal cations, *e.g.*, Zr and Hf.^15–18^ Subsequently, Zr(iv)-containing MOFs with terephthalate linkers (UiO-66 and UiO-66-NH_2_) composed of a Zr_6_(μ_3_-O)_4_(μ_3_-OH)_4_ core possess strong Zr–O bonds, which contribute to the exceptional stability of these MOFs under various conditions. In UiO-66-NH_2_, the polyhedron edges of the core are further connected by NH_2_-terephthalate linkers, forming a 12-connected Zr_6_(μ_3_-O)_4_(μ_3_-OH)_4_(NH_2_–COO)_12_ cluster that possesses high chemical and thermal stability, porosity, stability towards hydrolysis and ease of synthesis.^19–21^ This exceptional stability allows for the functionalization of the channels *via* post-synthetic modification (PSM) under various chemical conditions by tuning the cavity size.^22–26^

The dependence of our society on electronic appliances has extensively increased the requirement for energy sources. The limited supply of non-renewable energy sources has created a surge in the development of alternative energy sources. In this context, nuclear energy possesses several advantages in terms of the exclusion of combustion, which otherwise contributes to global warming.^27–31^ Nuclear reactors use uranium (one of the isotopes) as fuel; thus, the efficient recovery of uranium from different waste streams is essential. Hitherto, various liquid–liquid extraction systems^31–33^ and solid phase extractants (SPEs)^34^ have been utilized for the extraction/recovery of uranyl ions from waste matrices by extraction techniques such as ion exchange, solvent extraction, foam separation, co-precipitation, biomass collection, and sorption. However, SPEs are advantageous over solution-based extractants in terms of their insolubility in aqueous medium, dispensable organic solvents, quick response time, high sorption capacity, selectivity, stability, cost-effectiveness and environmental friendliness.

The SPE consists of a solid matrix grafted with a chelating group which binds to the target ions. Various SPE porous materials with large specific surface areas, including graphene, carbon-based materials, carbon nanotubes, polymer-based materials, magnesium hydroxide-based materials and mesoporous silica, have proven to be potential sorbents for uranium extraction.^35–46^ However, the major drawbacks associated with these materials are their irregular pore sizes, low surface areas, and hydrophobicity, which provide low sorption capacity for uranyl ions.

Compared to conventional SPEs, MOFs are three-dimensional materials that possess tunable pore sizes, high porosity, thermal/chemical stability, and reusability; thus, they are outstanding candidates for SPEs.^47–59^ In this context, the Zr(iv)-containing MOF UiO-66-NH_2_, with a BET surface area of 1112 m^2^ g^−1^, provides an excellent platform for the construction of MOFs with desired functionalities *via* PSM. Suitable functional groups serving as coordination sites can be anchored on UiO-66-NH_2_ (serving as micro-reactors) *via* covalent grafting for uranium extraction.^60^ In PSM, the porosity of the MOF allows reactive species to diffuse into the crystal structure and come in contact with the functional groups grafted to the framework.

**Scheme 1:**
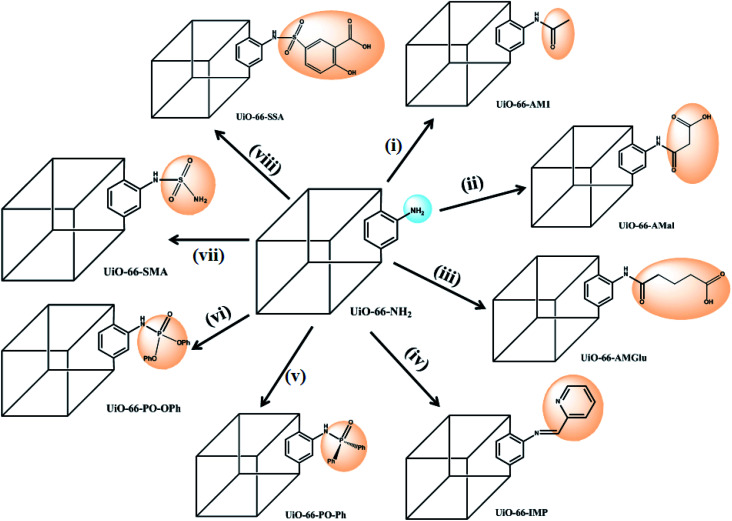
Post-synthetic modification (PSM) of UiO-66-NH_2_ with various functionalities: (i) acetic anhydride;^61^ (ii) maleic anhydride;^61^ (iii) glutaric anhydride;^62^ (iv) 2-pyridine carboxaldehyde;^63^ (v) Ph_2_POCl;^64^ (vi) Ph_2_O_2_POCl;^64^ (vii) sulfamic acid (NH_2_SO_3_H), EtOH, 80 °C, 24 h; (viii) 5-sulfosalicylic acid, EtOH, 80 °C, 24 h.

In the present study, we report the synthesis and characterization of eight MOFs covalently grafted *via* PSM and functionalized with different groups (Scheme 1), *viz.* amide (with/without pendant carboxylic acid), iminopyridine, phosphinic amide and sulphur-containing functionalities, to produce a library of eight different UiO-66-NH_2_ derivatives. The functionalized MOFs were subsequently employed as new SPE materials for uranium(vi) extraction *via* batch processes, providing a direct comparison of the sorption efficiencies of different functional groups on a common solid support. The influence of various analytical parameters, such as the pH, contact time, and desorption process, was investigated. A phosphorous-functionalized material, UiO-66-PO-Ph, exhibited the best sorption capacity in acidic conditions, while UiO-66-NH_2_ and iminopyridine-functionalized UiO-66-IMP showed excellent sorption in neutral conditions. Amide-containing MOFs UiO-66-AM1, UiO-66-AMMal and UiO-66-AMGlu, sulfur-based MOFs UiO-66-SMA and UiO-66-SSA, and phosphorous-functionalized UiO-66-PO-OPh displayed maximum sorption in basic conditions. The functionalized MOFs can be employed as SPEs in acidic/neutral/basic conditions; thus, they are suitable materials for uranium(vi) sorption.

## 2 Experimental section

### 2.1 Materials

All the chemicals and solvents were obtained commercially and were used as received without any further purification. ZrCl_4_ was procured from Acros Organics; 2-aminoterephthalic acid, glutaric anhydride, 2-pyridinecarboxaldehyde, diphenylphosphinic chloride and diphenylphosphoryl chloride were received from Sigma-Aldrich. Acetic anhydride was obtained from Reechem Pvt. Ltd. Maleic anhydride, sulfamic acid and 5-sulfosalicylic acid were received from Loba Chemie Pvt. Ltd. Triethylamine, acetonitrile, DMF, toluene and chloroform were obtained from Merck, while methanol and ethanol were purchased from SD Fine-Chem Ltd. and Changshu Hongsheng Fine Chemicals Co. Ltd., respectively.

### 2.2 Characterization

Infrared spectra of UiO-66-NH_2_ and the functionalized MOFs were recorded (KBr disk, 400–4000 cm^−1^) using an ABB MB3000 Fourier-transform infrared (FTIR) spectrometer. The powder XRD patterns were obtained using a GNR instrument with Cu Kα radiation (*λ* = 1.540598 Å) with a scan rate of 0.05° s^−1^ at 293 K. The BET surface area was measured at 77 K on a Autosorb iQ Station-1 after pretreatment by heating the samples under vacuum at 105 °C for 24 h. Thermogravimetric analysis was carried out in argon atmosphere on QMS 403 D NETZSCH (at a heating rate of 10 °C min^−1^). NMR spectral data were recorded from a Bruker FT NMR spectrometer (500 MHz for ^1^H) in DMSO-*d*_6_. The solution absorption spectra at room temperature were recorded on a UV-2100 UV-Vis-NIR spectrophotometer (Shimadzu) to analyze the metal complexes of uranyl ions. The pH of the solutions was measured using a Systronics 335 digital pH meter. The SEM images were analyzed using a Carl Zeiss Crossbeam 340 SEM, while energy dispersive X-ray (EDX) data were obtained using an Oxford Instruments X-Max^N^ detector. ChemBioDrawUltra 11.0 was used to generate graphics.

### 2.3 Synthesis

#### 2.3.1 Synthesis of UiO-66-NH_2_

UiO-66-NH_2_ was synthesized by following the literature procedure by Cohen *et al.*

### 2.4 PSM of UiO-66-NH_2_

#### 2.4.1 UiO-66-AM1 and UiO-66-AMMal

The synthesis was carried out by following the literature procedure by Cohen *et al.* for UiO-66-NH_2_.^61^

#### 2.4.2 UiO-66-AMGlu

The synthesis was carried out by following a literature procedure by Rassaei *et al.* developed for NH_2_-MIL-53 (Al and Cr) with minor modifications.^62^ UiO-66-NH_2_ (500 mg) was suspended in acetonitrile (50 mL) and stirred, followed by addition of glutaric anhydride (200 mg, 1.75 mmol). The reaction mixture was refluxed for 24 h and the resulting yellow powder was filtered, washed with acetonitrile (three times), and dried at 100 °C overnight. IR (KBr, cm^−1^): 3354 (br), 1704 (m), 1574 (vs), 1428 (vs), 1375 (s), 1297 (w), 1255 (w), 1159 (w), 771 (m), 667 (m), 527 (w).

#### 2.4.3 UiO-66-IMP

The synthesis was carried out by following a literature procedure by Yaghi *et al.* developed for (Zn_4_O)_3_(BDC-NH_2_)_3_(BTB)_4_ MOF with minor modifications.^63^ UiO-66-NH_2_ (500 mg) was immersed in a solution of 2-pyridinecarboxaldehyde (0.75 mL) in toluene (20 mL) and allowed to stand unperturbed for 5 days. The resulting yellow powder was washed thoroughly with CH_2_Cl_2_ and dried at 100 °C for 24 h. IR (KBr, cm^−1^): 3454 (br), 1697 (w), 1571 (vs), 1497 (w), 1434 (s), 1382 (vs), 1256 (m), 768 (m), 664 (m), 575 (w).

#### 2.4.4 UiO-66-PO-Ph and UiO-66-PO-OPh

The synthesis was carried out by following a literature procedure by Kapteijn *et al.* developed for NH_2_-MIL-53(Al) with minor modifications.^64^ UiO-66-NH_2_ (500 mg) was cooled to 0 °C with an ice bath in a two-neck round bottom flask with a magnetic stirring bar, and 8.83 mmol of Ph_2_POCl (1.5 mL)/(PhO)_2_POCl (1.7 mL) was added. The bath was removed after 30 min and diluted with acetonitrile (20 mL), and the mixture was stirred at room temperature for 24 h. The mixture was again cooled to 0 °C, followed by addition of triethylamine (0.46 mL, 3.32 mmol). The reaction temperature was raised to reflux (82 °C) and the reaction was maintained at this temperature for 24 h. After cooling to room temperature, the mixture was filtered, washed thoroughly with CH_3_CN and dried at 100 °C in an oven for 24 h. UiO-66-PO-Ph; IR (KBr, cm^−1^): 3372 (br), 3060 (w), 1686 (w), 1619 (w), 1571 (vs), 1501 (w), 1497 (w), 1434 (vs), 1386 (vs), 1256 (m), 1131 (s), 1042 (s), 1019 (s), 760 (m), 731 (m), 690 (m), 660 (m), 553 (s). UiO-66-PO-OPh; IR (KBr, cm^−1^): 3397 (br), 2965 (m), 2939 (m), 2741 (w), 2671 (s), 2490 (w), 1575 (s), 1490 (s), 1430 (vs), 1386 (vs), 1201 (s), 1090 (s), 1038 (w), 945 (m), 760 (s), 690 (w), 656 (s), 530 (m).

#### 2.4.5 UiO-66-SMA and UiO-66-SSA

UiO-66-NH_2_ (500 mg) was suspended in an ethanolic solution (40 mL) of 9.96 mmol of sulfamic acid (0.97 g)/5-sulfosalicylic acid dihydrate (2.53 g) and refluxed for 24 h. The reaction mixture was filtered and washed with ethanol (3 × 20 mL), followed by soaking in fresh ethanol (30 mL) for 24 h and drying at 100 °C for 24 h. UiO-66-SMA; IR (KBr, cm^−1^): 3407 (br), 1625 (w), 1566 (vs), 1499 (w), 1428 (vs), 1384 (vs), 1250 (m), 1148 (br), 1061 (br), 1001 (br), 966 (br), 911 (br), 768 (m), 662 (m), 579 (w). UiO-66-SSA; IR (KBr, cm^−1^): 3420 (br), 1588 (s), 1501 (w), 1427 (vs), 1382 (vs), 1254 (m), 1213 (w), 1164 (m), 1085 (w), 1032 (s), 830 (w), 800 (w), 766 (m), 665 (s), 593 (m).

**Caution!** Diphenylphosphinic chloride and diphenylphosphoryl chloride cause irritation and a burning sensation upon contact with skin; hence, they should be handled with proper covering and care.

### 2.5 Sorption experiments

The sorption experiments with uranyl ions were performed using the batch technique in glass equilibration tubes with uranium stock solution (50.1 mg mL^−1^) prepared by dissolving UO_2_(NO_3_)_2_·6H_2_O in 0.1 M HNO_3_. Feed solutions of uranium (500 μg mL^−1^) with different pH values (1 to 9) were prepared from uranium stock solution (50.1 mg mL^−1^), and the pH of the solutions was optimized by addition of deionized water and very small volumes of 0.1 M HNO_3_/NaOH solutions. In all sorption experiments, an uranium solution with a given concentration, pH value and quantity was added to a glass tube containing MOFs (10 mg). Experiments with varying contact times (5 minutes to 4 h) were also carried out at the optimized pH of the uranyl solution. The suspension was then agitated in a shaker at room temperature for a required time, and the solid was allowed to settle by centrifugation for 10 min (5000 rpm) at 25 °C. The concentration of uranyl ions in the supernatant solution was measured by a spectrophotometric method using Arsenazo III as the chromogenic agent; the absorption of the complex was monitored at 655 nm.

The amount of uranyl sorption, *q*_e_ (mg U g^−1^), at equilibrium time *t* was calculated by:1
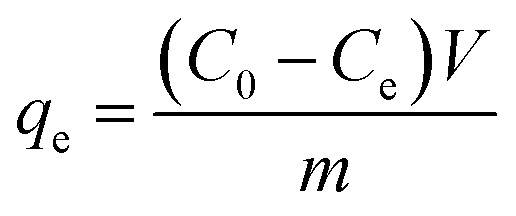
where *q*_e_ (mg U g^−1^) is the quantity of uranium adsorbed on 1 g of MOF in time *t* (h), *C*_0_ and *C*_e_ denote the initial and equilibrium concentrations (mg L^−1^) of uranium, respectively, *V* is the volume of the solution (L), and *m* represents the mass of the MOF (g).

The sorption efficiency of uranyl ions *via* sorption from aqueous solution was calculated by the following equation:2
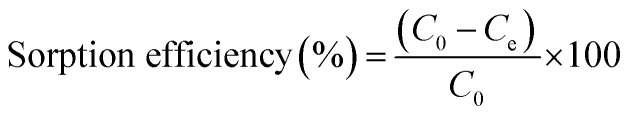


The distribution coefficient, *K*_d_, to measure the sorption capability of the sorbent was calculated as:3
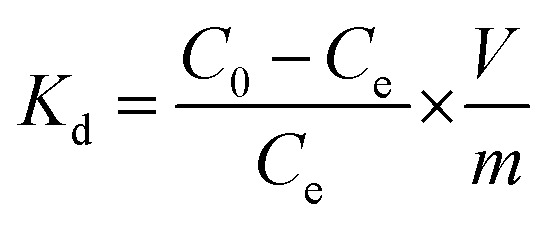


### 2.6 Desorption studies

In order to evaluate the reversibility of uranium sorption on the MOFs, desorption studies were carried out for the uranyl ions sorbed on the MOFs (UiO-66-NH_2_, UiO-66-IMP and UiO-66-PO-Ph) using different eluents. The uranyl ions sorbed on the MOFs surfaces were desorbed by suspending each uranium-loaded MOF in 3 mL solutions of different eluents, *e.g.* deionized water, 0.01 M HNO_3_, 0.1 M HNO_3_, 0.01 M HCl, 0.1 M HCl, 0.01 M Na_2_CO_3_ or 0.1 M Na_2_CO_3_, for a period of 4 h. Each sample was then collected by centrifugation and the concentration of uranyl ions in the supernatant was analyzed using spectrophotometry, as mentioned earlier.

### 2.7 Recyclability studies

The reusability of MOFs for uranium sorption was tested by recycling experiments. The sorption of uranium (500 ppm) on the MOFs (10 mg in 3 mL) was first carried out for 4 h at 25 °C to reach equilibrium, followed by centrifugation and removal of the supernatant for analysis of the residual uranium. The filtered solid was thoroughly washed with deionised water and dried at 100 °C. Thereafter, the uranium loaded on the MOFs was removed by desorption experiments using 3 mL of eluent and was stirred for 4 h, followed by centrifugation and separation of the supernatant for analysis. Subsequently, the stability of the MOFs was analyzed by powder XRD, and the experiment was repeated for two cycles to study the recyclability of MOFs for uranium sorption.

## 3 Results and discussion

### 3.1 Post-synthetically modified MOFs

The PSM of MOFs based on linker modification *via* covalent grafting of new functional moieties allows the creation of new materials with different functionalizations and properties.^22–26^ This route also proves to be advantageous because the requirement of specific and convoluted linkers becomes dispensable for the construction of targeted MOFs. Therefore, it becomes possible to modify the linkers that are already present *via* reacting them with various organic groups, resulting in MOFs that possess desired applications.

The presence of pendant amino groups in the NH_2_-BDC linker in the MOFs has been proven to be excellent for anchoring desired organic functionalities to the framework *via* PSM, thereby providing reaction space within the MOFs and leading to modification of the cavities for desired applications.^60^ Cohen and co-workers reported functionalization of UiO-66-NH_2_ with four anhydrides *via* covalent PSM to generate amide-functionalized frameworks.^61^ Rassaei and co-workers extended the PSM of NH_2_-BDC-appended MOFs by anchoring glutaric anhydride on NH_2_-MIL53(Al) and NH_2_-MIL101(Cr) to produce pendant carboxylic acids, which were further employed to covalently couple the enzyme glucose oxidase.^62^ In another study by Yaghi and co-workers, a metal binding site was introduced using NH_2_-BDC as the linker in the MOF (Zn_4_O)_3_(BDC-NH_2_)_3_(BTB)_4_, pointing to the cage centers; upon PSM, an iminopyridine chelating assembly was formed, followed by metalation to produce a Pd(ii) metal-complexed MOF.^63^ In work reported by Kapteijn and co-workers, NH_2_-MIL-53(Al) was used to anchor the diphenylphosphinyl (Ph_2_PO-) moiety bearing a phosphinic amide functional group, which in turn stabilized the large pores of the MOF and also changed its optical properties due to the electronic influence of the PO group.^64^

The success of PSM carried out in MOFs possessing NH_2_-BDC linkers as well as the porosity and stability of UiO-66-NH_2_ inspired to incorporate suitable organic functionalities within MOFs. The functionalized MOFs can be further utilized as reaction spaces to bind uranyl ions for their effective extraction from aqueous waste streams. The PSM of UiO-66-NH_2_ was carried out by reaction of the NH_2_-BDC motif with various organic molecules, including various anhydrides,^61,62^ 2-pyridinecarboxaldehyde,^63^ and phosphorous-^64^ and sulphur-based functionalities (Scheme 1), producing new MOFs that show completely different uranium sorption behaviors compared to the parent MOF (UiO-66-NH_2_). Additionally, the post-synthetically modified MOFs were found to be sufficiently robust, possessing accessible channels to permit binding of uranyl ions with the chelating moieties.

The reaction of UiO-66-NH_2_ with anhydrides introduces an amide moiety in the framework, UiO-66-AM1 (acetic anhydride), in addition to free carboxylate groups, UiO-66-AMMal (maleic anhydride) and UiO-66-AMGlu (glutaric anhydride), which otherwise cannot be readily obtained by direct solvothermal synthesis. Additionally, the introduction of metal binding sites in UiO-66-NH_2_ was incorporated by covalently bound iminopyridine, forming UiO-66-IMP (2-pyridinecarboxaldehyde) to provide chelating ligands for uranyl binding.

In spite of the formation of stronger bonds with metal atoms, reports of MOFs with phosphorous-based moieties are scarce compared to those with carboxylates.^65^ The phosphorous-based MOFs are also found to exhibit good thermal stability and insolubility even in strong acid solution; thus, they are suitable candidates for solid supports for extraction purposes. However, the main drawback of phosphorous-based MOFs lies in their lack of permanent porosity; this can be overcome by the PSM concept, which produces phosphorous-based porous MOFs. Therefore, two new phosphorous-grafted UiO-66-NH_2_ derivatives were synthesized, *viz.* UiO-66-PO-Ph (diphenylphosphinic chloride) and UiO-66-PO-OPh (diphenylphosphoryl chloride).

Similarly, despite the versatile binding modes and stability offered by sulfur-based ligands compared to carboxylate-based MOFs, only a handful of sulfur-based MOFs have been reported to date.^66,67^ This can primarily be attributed to their weaker coordination with transition metals as well as their formation of inferior porous frameworks relative to carboxylate MOFs. However, PSM can also prove to be an efficient tool to construct stable MOFs by incorporating sulfur-based moieties inside the porous framework built from carboxylate-based linkers. With this idea, sulfur-based groups, *i.e.* sulfamic acid (UiO-66-SMA) and 5-sulfosalicylic acid (UiO-66-SSA), were incorporated in UiO-66-NH_2_ for the construction of two new MOFs.

### 3.2 Characterization of post-synthetically modified MOFs

**Fig. 1:**
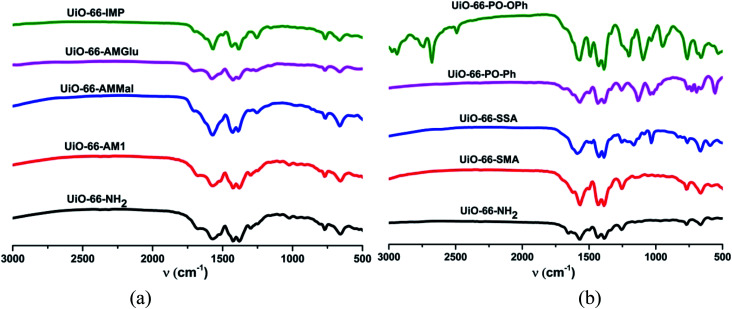
FTIR spectra of UiO-66-NH_2_ and different functionalized MOFs.

The FT-IR spectra of UiO-66-NH_2_ post-synthetically modified *via* anhydride functionalization to produce MOFs containing amide groups, *viz.* UiO-66-AM1, UiO-66-AMMal and UiO-66-AMGlu, exhibit a weak peak at around 1700 cm^−1^; this is attributed to the C=O moiety of the amide functionality (Fig. 1a). The iminopyridine unit incorporated in UiO-66-IMP exhibits a sharp peak at *ca.* 1600 cm^−1^, corresponding to imine formation (C=N) (Fig. 1a). The sulphur-containing MOFs, UiO-66-SMA and UiO-66-SSA, display peaks around 1100 to 1200 cm^−1^, which are attributed to S=O stretching (Fig. 1b). The functionalization with phosphorous-containing organic groups led to incorporation of phosphinic amide groups (–NH–P=O) in UiO-66-PO-Ph and UiO-66-PO-OPh; this is evidenced by the characteristic stretching vibrations of P=O observed at 1142 cm^−1^ and 1201 cm^−1^, respectively, while UiO-66-PO-OPh also shows P–O–C stretching at 1090 cm^−1^ (Fig. 1b).

**Fig. 2:**
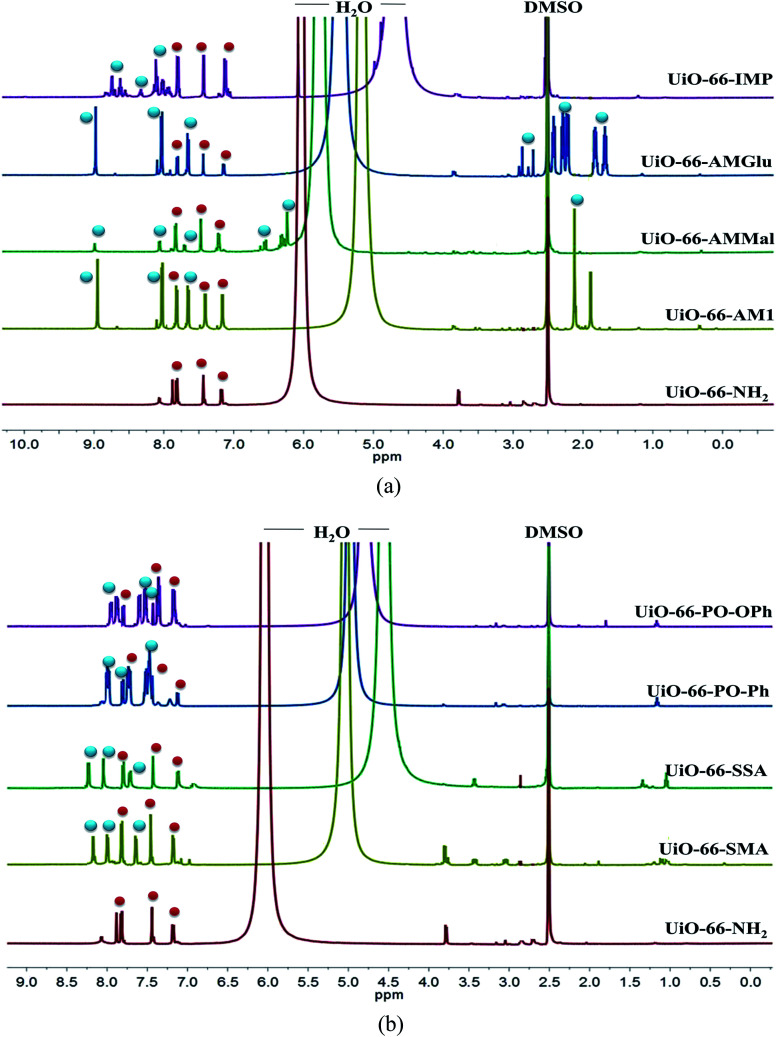
500 MHz ^1^H-NMR spectra (DMSO-*d*_6_) of UiO-66-NH_2_ and different functionalized MOFs. Red spheres represent unmodified NH_2_-BDC and blue spheres present the modified NH_2_-BDC ligands.

The PSM of UiO-66-NH_2_ was further confirmed by acid-digested (HF and DMSO-*d*_6_) NMR spectroscopy (^1^H-, ^13^C- and ^31^P-NMR) for all MOFs. The ^1^H-NMR spectra of all PSM MOFs with the modified BDC ligand exhibit a downfield shift for the aromatic protons compared to the parent MOF (Fig. 2a and b). The ^1^H-NMR spectra of UiO-66-AM1 and UiO-66-AMMal match well with those reported in the literature, thereby confirming the formation of functionalized MOFs.^61^ The acid-digested UiO-66-AMGlu predominantly displayed peaks corresponding to 2-amino-1,4-benzene dicarboxylic acid, along with new peaks corresponding to the alkyl groups of glutaric anhydride appearing in the range of 1.6–2.9 ppm. The iminopyridine unit in UiO-66-IMP exhibits peaks attributed to the iminopyridine moiety in the aromatic region, and the modified BDC ligand shows a downfield shift. The sulphur-modified MOFs, UiO-66-SMA and UiO-66-SSA, showed a downfield shift due to modified BDC. The phosphinic amide groups in UiO-66-PO-Ph and UiO-66-PO-OPh showed downfield shifts for the aromatic protons of BDC in addition to the aromatic protons of the new phenyl rings. The ^13^C-NMR spectra also exhibit new peaks corresponding to the modified frameworks and confirming the presence of the functionalities introduced in the framework (Fig. S1 and S2†). In addition, the ^31^P-NMR spectra of UiO-66-PO-Ph and UiO-66-PO-OPh match well with those of the starting materials, *i.e.* diphenylphosphinic chloride and diphenylphosphoryl chloride, respectively (Fig. S3 and S4†). Therefore, the NMR studies also confirmed the conversion of UiO-66-NH_2_ to modified MOFs, and the percentages of conversion are noted in Table S1.†

**Fig. 3:**
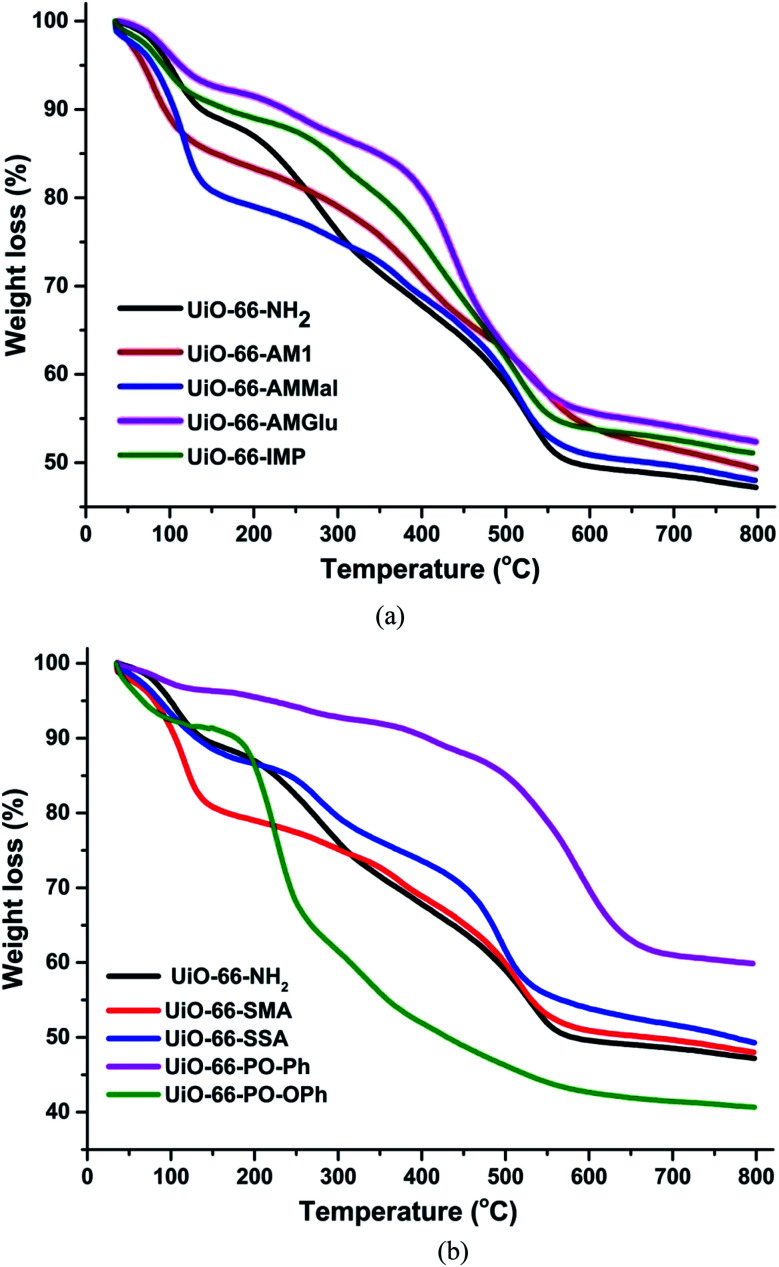
TGA plot of UiO-66-NH_2_ and different functionalized MOFs.

The thermogravimetric analysis of UiO-66-NH_2_ and other functionalized MOFs varied with different functional groups (Fig. 3a and b). The TGA profiles of UiO-66-NH_2_, UiO-66-AM1 and UiO-66-AMMal were consistent with literature values, while UiO-66-Glu and UiO-66-IMP exhibited TGA profiles comparable to that of the parent MOF. However, the sulfur-based MOF UiO-66-SMA exhibits lower stability compared to the parent MOF and shows a gradual weight loss at around 150 °C, possibly due to decomposition of the functionalized ligand. On the other hand, UiO-66-SSA shows enhanced stability compared to UiO-66-SMA and is stable up to 300 °C, after which gradual weight loss was observed. Interestingly, the phosphorous-based UiO-66-PO-Ph showed an enhancement in the thermal stability compared to the parent MOF and is stable up to 500 °C. The TGA profile of UiO-66-PO-OPh is similar to that of the parent MOF and shows initial gradual weight loss after 180 °C. All TGA profiles displayed decomposition of the framework at around 440 °C to 500 °C, probably due to the formation of ZrO_2_.

In general, MOFs display appreciable thermal stability on account of their multiple electrostatically stabilized metal–ligand bonds. However, the thermal decomposition depends on the metal and ligand groups. The initial weight loss in UiO-66-NH_2_ occurs due to irreversible ligand-based decarboxylation, leading to collapse of the framework. However, in the case of UiO-66-PO-Ph, the high stability is due to the stronger bonds displayed by phosphorous-based moieties. However, the lower stability of UiO-66-PO-OPh can be attributed to the presence of –O– spacers in the framework; their flexible nature leads to easy dissociation of the ligand. Moreover, the enhanced stability of UiO-66-SSA compared to UiO-66-SMA is probably due to their differences, *i.e.* the presence of aromatic rings in the former while the latter contains only aliphatic groups.

Moreover, the functionalization of UiO-66-NH_2_ with various moieties was confirmed by SEM-EDX measurements of the solid samples to analyze the quantitative functionalization of UiO-66-NH_2_ (Fig. S5–S13†).

Surface area analysis of all the reported MOFs was also carried out in order to examine the effects of functionalization on the porosity of UiO-66-NH_2_ by Brunauer–Emmett–Teller (BET) measurements with N_2_ adsorption at 77 K (Table S2†). As observed by Cohen *et al.*,^61^ the PSM of UiO-66-NH_2_ results in a decrease in the porosity after functionalization with different organic moieties, where larger functional groups lead to a greater decrease in pore size, thereby reducing the porosity of the MOF.

**Fig. 4:**
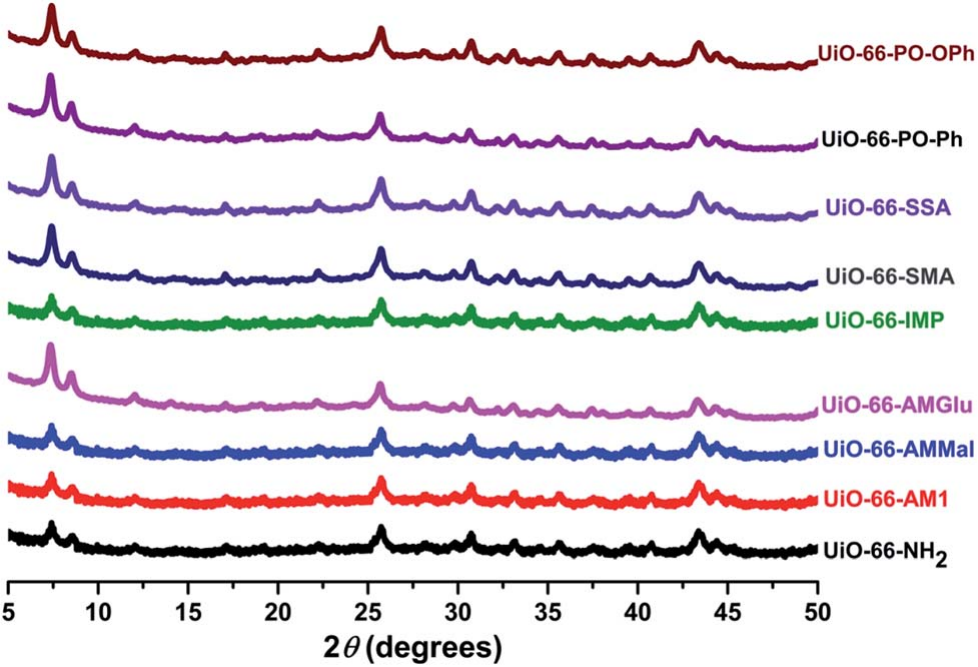
PXRD patterns of UiO-66-NH_2_ and the different functionalized MOFs.

The powder-XRD patterns of UiO-66-NH_2_ and the functionalized MOFs were compared and showed that the PSM occurred without causing any degradation of the parent framework (Fig. 4). However, minor changes were observed, which can be attributed to the variations in the electron density caused by the presence of functionalized ligands.

### 3.3 Sorption studies

In the recent past, employment of MOFs as solid sorbents has been reported for the extraction of uranyl ions due to their higher surface areas, larger pore diameters, and chemical and thermal stability. Tang and co-workers reported the uranyl sorption behavior of UiO-66 and UiO-66-NH_2_ in aqueous solution; they reported a sorption capacity of more than 100 mg g^−1^ in *ca.* 4 h at pH 5.5.^58^ Inspired by the excellent sorption behavior of UiO-66-NH_2_, our strategy involved functionalization of the pendant –NH_2_ groups in UiO-66-NH_2_ and covalent grafting with various functional groups. These anchored groups were then evaluated and found to effectively bind with uranyl ions, thereby improving the sorption capacity of the parent MOF UiO-66-NH_2_. In addition, the new incoming groups may alter the stability of the MOF in acidic/basic media; thus, UiO-66-NH_2_ is a versatile MOF in the extraction of radionuclides operating at various pH values. This motivated us to evaluate the sorption behavior of the functionalized UiO-66-NH_2_ MOFs as sorbents for extraction of uranyl ions from acidic and basic media; this was studied by varying the analytical parameters, including the pH, contact time, and desorption process.

### 3.4 Influence of pH on the sorption of uranyl ions

The pH of the medium plays a crucial role in the sorption of uranyl ions because it influences the solubility and speciation of uranyl ions as well as the charge on the functional groups. Therefore, batch analysis was carried out over the pH range from 1 to 9. As reported by Tang and co-workers, the sorption of uranyl ions was maximum at pH 5.5 for UiO-66-NH_2_; we attempted to observe the sorption in basic solutions as well. It was interesting to observe that UiO-66-NH_2_ displayed maximum sorption efficiency (91%) at pH 7. However, the uranyl sorption efficiency of the functionalized UiO-66-NH_2_ MOFs was found to vary with the pH of the solution (Table 1) (Fig. S14 and 15†). The amide-functionalized MOFs, *viz.* UiO-66-AM1 (40%), UiO-66-AMMal (31%), and UiO-66-AMGlu (70%), exhibited maximum sorption in basic conditions (pH 8). The sulfur-based MOFs, *viz.* UiO-66-SMA (65%) and UiO-66-SSA (27%), also exhibited maximum sorption in basic solution (pH 8). However, the iminopyridine-containing UiO-66-IMP (90%), similar to the parent UiO-66-NH_2_, displayed maximum extraction in neutral solution (pH 7). Interestingly, the phosphorous-containing MOF UiO-66-PO-Ph (96%) displayed maximum sorption in acidic solution (pH 3), while UiO-66-PO-OPh (50%) showed maximum sorption in basic solution (pH 8).

**Table 1:** Sorption of uranyl ions on UiO-66-NH_2_ and its derivatives

MOF	Sorption%	pH	*q*_e_	*K*_d_
UiO-66-PO-Ph	96	3	111.9	7460
UiO-66-NH_2_	92	7	87.5	3244
UiO-66-IMP	90	7	85.7	2434
UiO-66-AMGlu	70	8	57.2	600
UiO-66-SMA	65	8	50.5	482
UiO-66-PO-OPh	50	8	42.5	279
UiO-66-AM1	40	8	30.1	194
UiO-66-AMMal	31	8	26.8	97
UiO-66-SSA	27	8	24.1	90

**Fig. 5:**
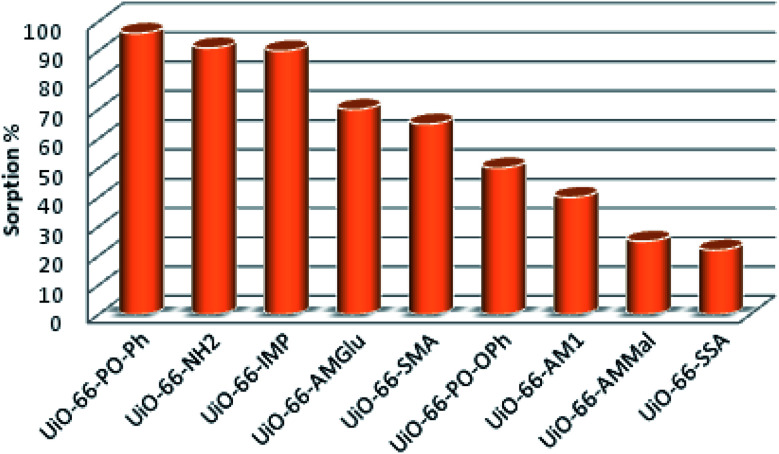
Uranyl ion sorption% of UiO-66-NH_2_ and the different functionalized MOFs.

**Fig. 6:**
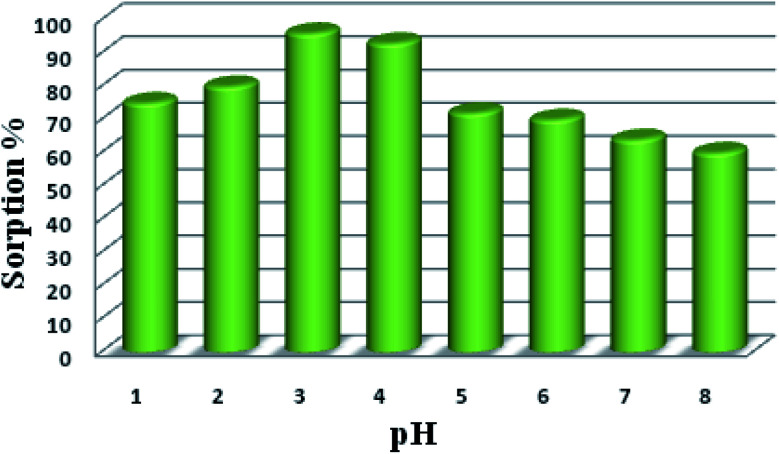
Effects of pH variation on uranyl ion sorption onto UiO-66-PO-Ph: *t* = 120 min, *m*_sorbent_ = 10.0 mg, *V*_solution_ = 3 mL, *C*_0_ = 500 mg L^−1^, *T* = 25 ± 0.5 °C.

The sorption efficiency of MOFs follows the order UiO-66-PO-Ph (96%) > UiO-66-NH_2_ (91%) > UiO-66-IMP (90%) > UiO-66-AMGlu (70%) > UiO-66-SMA (65%) > UiO-66-PO-OPh (50%) > UiO-66-AM1 (40%) > UiO-66-AMMal (31%) > UiO-66-SSA (27%) (Fig. 5). Moreover, the amount of uranyl sorption, *q*_e_ (mg U g^−1^), at equilibrium time *t* on the surface of the MOFs was varied by changing the functional group of UiO-66-NH_2_ (Table 1). The parent MOF, UiO-66-NH_2_, shows uranyl sorption of 87.1 mg g^−1^ at pH 7; meanwhile, UiO-66-PO-Ph showed significantly high uranyl ion sorption (111.9 mg g^−1^) at pH 3, which is the highest among UiO-66-NH_2_ and the other functionalized UiO-66-NH_2_ frameworks (Table 1). Moreover, the *q*_e_ values for UiO-66-PO-Ph over the pH range of 1–8 varied from 24.1 to 111.9 mg g^−1^ (Fig. 6).

The distribution coefficient, *K*_d_, measures the sorption capability and effectiveness of a sorbent at a particular concentration, and a higher *K*_d_ value is regarded to indicate effective sorption of the target species. The *K*_d_ value was excellent for UiO-66-PO-Ph (7460), two-fold that of the parent UiO-66-NH_2_ (3244) and threefold that of UiO-66-IMP (2434) (Table 1). The *K*_d_ values for these three MOFs, *viz.* UiO-66-PO-Ph, UiO-66-NH_2_, and UiO-66-IMP, are in the acceptable range (>500). Due to the excellent *K*_d_ value of UiO-66-PO-Ph (7460), it is an excellent sorbent material for uranyl ion extraction. These results exhibit that the difference in U sorption by UiO-66-NH_2_ and other functionalized MOFs with variation in pH of the solution can be attributed to the different functional groups and surface areas of these MOFs.

pH plays a crucial role in the sorption of U(vi) ions because of the pH-induced protonation (lower pH) and deprotonation (higher pH) of the functionalities grafted on UiO-66-NH_2_. Moreover, the coordination or hydrogen bond interactions between the anchored functional moieties and incoming U(vi) ions incite an increase of the sorption capacity. In addition, pH-induced U(vi) speciation may be an important factor for pH-dependent sorption. The major hydrolysed complex ions in solution are UO_2_^2+^ at pH 2–5 and UO_2_^2+^, [UO_2_(OH)]^+^, [(UO_2_)_3_O(OH)_3_]^+^, [(UO_2_)_2_(OH)_2_]^2+^ and [UO_3_(OH)]^5+^ at pH 5–8.5. These ions exhibit affinity towards the functional groups present on UiO-66-NH_2_.

### 3.5 Effects of contact time: sorption kinetics

**Fig. 7:**
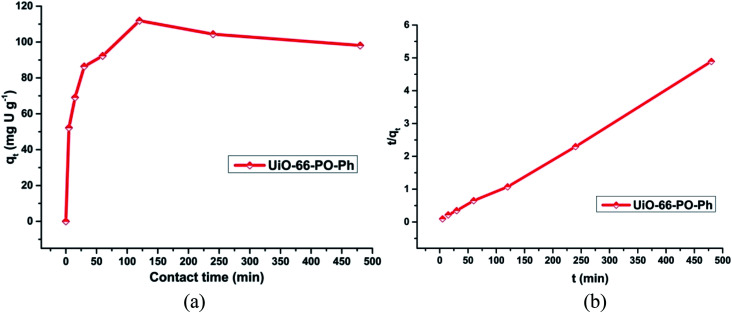
(a) Effect of contact time on uranyl ion sorption onto UiO-66-PO-Ph; (b) *t*/*q*_*t*_*versus t* plot for UiO-66-PO-Ph; pH = 3, *m*_sorbent_ = 10.0 mg, *V*_solution_ = 3 mL, *C*_0_ = 500 mg L^−1^, *T* = 25 ± 0.5 °C.

In order to evaluate these MOF sorbents for practical applications, the sorption kinetics of uranyl sorption on UiO-66-NH_2_ and the other functionalized MOFs as well as the rates of uranyl ion sorption by the MOFs (0.010 g) were studied with an initial concentration of uranyl ion of about 500 ppm (3 mL) for 0, 5, 15, 30, 60, 120, 240 and 480 min (Fig. S16–19†) (Fig. 7a). The study was carried out at pH 3 (UiO-66-PO-Ph), 7 (UiO-66-NH_2_ and UiO-66-IMP) or 8 (UiO-66-AM1, UiO-66-AMMal, UiO-66-AMGlu, UiO-66-SMA, UiO-66-SSA and UiO-66-PO-OPh).

It was observed that the rate of uranium sorption in the MOFs showed an increment in the initial 2 h, after which the sorption process attained equilibrium at about 3 h. However, in the cases of UiO-66-AM1 and UiO-66-IMP, the sorption kinetics operated at a slower rate compared to the other MOFs, and they attained equilibrium in about 4 h. The variation in the sorption behavior of UiO-66-NH_2_ and the other functionalized MOFs clearly indicates the presence of suitable coordination interactions between the uranyl ions and the MOFs *via* the grafted functional moieties. In addition, this variation is probably caused by the variations in surface area and pore volume upon changing the functional group, thereby affecting the time taken by the uranyl ions to diffuse into the MOF framework. Moreover, the strength of the interactions of the MOFs with incoming uranyl ions affects the sorption of the ions in the MOFs.

The *t*/*q*_*t*_*versus t* plot shows linearity (Fig. S20†) (Fig. 7b), with a correlation coefficient of more than 0.98 (Table S3†), and the calculated *q*_e_ (slope) and *k*_2_ (intercept) match well with the experimental values. Thus, the pseudo-second order model explains the kinetics of uranyl sorption *via* UiO-66-NH_2_ and the other functionalized MOFs more appropriately. Moreover, this suggests that the mechanism of sorption is primarily controlled by the chemical interactions between uranyl ions and the MOF framework, not by physical sorption; thus, the diffusion of uranyl ions affects the rate of the reaction.

The initial increase in the sorption amount with time can be attributed to the availability of vacant sorption sites in the beginning. However, after attaining equilibrium, a decrease in sorption is observed; this can occur due to partial blockage of the pores of the MOF materials, which leads to a decrease in the total surface area and further decreases the diffusion of uranyl ions through the nano-channels.

The anchoring of organic functionalities onto UiO-66-NH_2_ was intended to create MOF materials with similar frameworks but variable porosities, surface areas and binding affinities, resulting in differences in sorption capacity. The difference in the extraction behavior of MOFs can be attributed to the variation in the space inside the MOF, the interaction of the organic moiety with uranyl ions with variable bond strengths, and the sorption efficiency in the presence of acids and bases.

However, we anticipate that the grafted functional groups will play major roles as binding sites in the MOF, providing variable electrostatic interactions with uranyl species and thereby changing the sorption behavior with changing pH while the other parameters remain the same (MOF dose, uranium concentration, and contact time). Interestingly, due to the high sorption capacity of UiO-66-PO-Ph, it is an excellent solid sorbent for the extraction of uranyl ions from acidic solution. Moreover, the higher level of sorption shown by UiO-66-PO-Ph compared to the parent MOF (UiO-66-NH_2_) indicates the participation of the grafted organic functionalities in binding uranyl ions within the channels. The motif for uranyl ion binding to phosphorous-based moieties is known to be monodentate coordination through the phosphonyl oxygen in UiO-66-PO-Ph rather than UiO-66-PO-OPh. The difference in sorption efficiency between UiO-66-PO-OPh and UiO-66-PO-Ph (with other factors remaining the same) clearly indicates the participation of the incorporated functional groups in binding the incoming U(vi) ions in the channels of the MOFs. The presence of the –O– spacer in UiO-66-PO-OPh may be a decisive factor because it provides more flexibility to the two phenyl rings; this can lead to variable geometry due to bending and rotation. This can further affect the phenyl ring arrangements; they will require more room for their accommodation, thereby leading to differences in binding to the U(vi) ions during sorption.

Amides and amines are known to bind to uranyl ions more efficiently than carboxylic groups,^32,34^ as observed in the case of the parent MOF UiO-66-NH_2_. The imine-containing UiO-66-IMP also exhibits excellent sorption efficiency (90%); this can be attributed to stronger interactions with the incoming uranyl ions *via* the imine and pyridine nitrogens, leading to enhanced sorption. On the other hand, the sulfur-containing MOF UiO-66-SMA exhibits almost double the sorption efficiency of UiO-66-SSA; this may be due to the less bulky grafted groups in the former, which afford more available surface area for the incoming uranyl ions. However, the amide-containing UiO-66-AM1 shows much lower sorption (40%). In addition, the MOFs containing both amide and carboxylic moieties exhibit sorptions ranging from 70% (UiO-66-AMGlu) to 31% (UiO-66-AMMal).

### 3.6 Desorption and recyclability studies

**Fig. 8:**
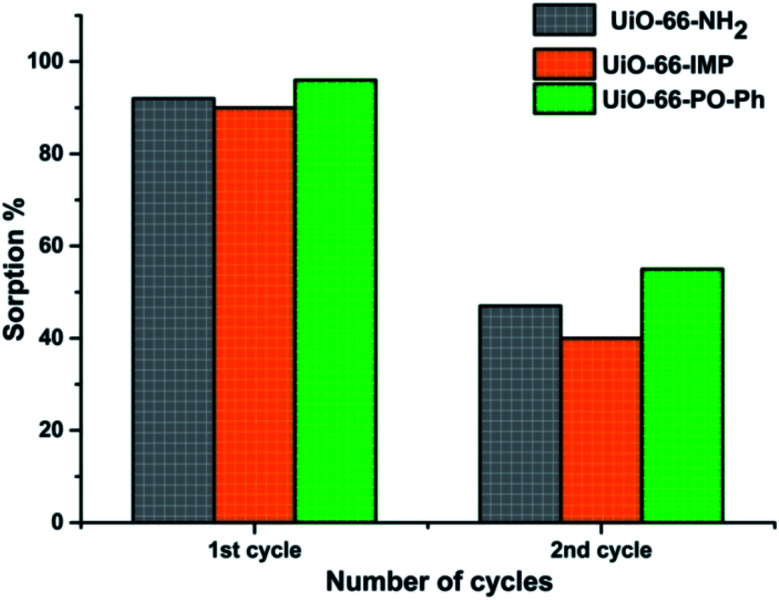
Recyclability tests for the MOFs.

The desorption studies of the adsorbed uranyl ions from the MOFs exhibiting nearly 90% sorption were carried out by suspending 10 mg of uranyl ions-sorbed MOFs in 3 mL of various eluent solutions (deionized water, 0.01 M HNO_3_, 0.1 M HNO_3_, 0.01 M HCl, 0.1 M HCl, 0.01 M Na_2_CO_3_ or 0.1 M Na_2_CO_3_) for 4 h at room temperature. The material was then collected by centrifugation, and the concentration of uranyl ions in the supernatant was analyzed as reported earlier. Successful recovery (*ca.* 90%) was obtained with 0.1 M HNO_3_ (UiO-66-PO-Ph) and 0.01 M HNO_3_ (UiO-66-NH_2_ and UiO-66-IMP). The experiments were repeated twice, for UiO-66-NH_2_, UiO-66-IMP and UiO-66-PO-Ph, in order to analyze the recyclability of these MOFs, after which the sorption capacity decreased (∼40–50%) (Fig. 8) which is attributed to the partial destruction of the MOF materials (Fig. S21–S23†). Further studies will be performed to improve the recyclability of the MOFs.

## 4 Conclusions

Herein, we report eight MOF materials that were functionalized by the PSM strategy of anchoring various functional groups, *viz.* amide (with/without pendant carboxylic acid), iminopyridine, phosphinic amide and sulphur. The functionalized MOF materials possessed similar frameworks but variable porosities, surface areas and binding affinities; this resulted in variations in sorption capacity towards uranyl ion extraction in acidic/neutral/basic conditions (pH 1 to 9). The variable space and electrostatic interaction of the decorated functionalized MOFs with the incoming uranyl species and the sorption efficiency in the presence of acids and bases played a major role in affecting the sorption behavior.

Interestingly, due to the enhanced thermal stability (∼500 °C) and highest sorption capacity of UiO-66-PO-Ph, it is an excellent solid state sorbent for the extraction/recovery of uranyl ions from acidic solution. The efficient binding of amides and amines/imines with uranyl ions was reflected in the case of the parent MOF UiO-66-NH_2_ and UiO-66-IMP, which also exhibit excellent sorption efficiency. Due to the moderate recyclability of UiO-66-NH_2_, UiO-66-IMP and UiO-66-PO-Ph, these materials are suitable for environmental cleanup.

This approach of facile preparation of MOFs as well as their grafting with suitable functional moieties will enable the development of new materials *via* covalent binding of analogous phosphorus/sulfur/amide/imine-containing functionalities to other MOFs bearing pendant moieties for efficient uranyl sorption. The strategy adopted herein can be further extended and improved to obtain promising alternatives for efficient uranyl ion sorption in acidic/neutral/basic medium as well as recyclability for radioactive waste removal for a greener environment.

## Conflicts of interest

There are no conflicts to declare.

## Supplementary Material

RA-010-D0RA00410C-s001
