# Supplementary material for: Assorted functionality-appended UiO-66-NH2 for highly efficient uranium(vi) sorption at acidic/neutral/basic pH
Source: RSC Adv. 2020 Apr 9;10(25):14650–61. doi: 10.1039/d0ra00410c (PMC9051904; doi:10.1039/d0ra00410c)
Supplement: RA-010-D0RA00410C-s001 [file RA-010-D0RA00410C-s001.pdf]

Electronic Supplementary Material (ESI) for RSC Advances.
This journal is © The Royal Society of Chemistry 2020

Supporting Information

Assorted Functionalities Appended UiO-66-NH_2 for Highly Efficient Uranium (VI) Sorption in Acidic/Neutral/Basic pH

Sarita Tripathi,ǂ‡ B. Sreenivasulu,ǂ A. Suresh,ǂ‡ C. V. S. Brahmmananda Raoǂ‡ and N. Sivaramanǂ‡**

ǂFuel Chemistry Division, Indira Gandhi Centre for Atomic Research, Kalpakkam, Tamil Nadu-603102, India

‡Homi Bhabha National Institute (HBNI)

*Email: sarita17aug@gmail.com

sivaram@igcar.gov.in

Table of Contents

Table S1: Conversion of UiO-66-NH_2 to different functionalized MOFs	S3
500 MHz ^{13}C-NMR spectra (DMSO-d_6) of UiO-66-NH_2 and different functionalized MOFs	S4
^{31}P NMR (600 MHz, ^{1}H decoupled) in DMSO-d_6	S5
SEM and EDX spectra of UiO-66-NH_2 and different functionalized MOFs	S6-S10
Table S2: BET surface area of UiO-66 and different functionalized MOFs	S11
Effect of pH on U(VI) sorption onto MOFs	S12
Effect of contact time on U(VI) sorption onto MOFs	S13-14
Kinetic study	S15-16
Powder XRD patterns of recyclability studies	S17

Table S1. Conversion of UiO-66-NH_2 to different functionalized MOFs

MOF	% Conversion
UiO-66-AM1	73.4
UiO-66-AMMal	33.3
UiO-66-AMGlu	54.1
UiO-66-IMP	56.2
UiO-66-SMA	68.7
UiO-66-SSA	50.2
UiO-66-PO-Ph	54.9
UiO-66-PO-OPh	43.1

The percent conversion of the amine groups in the UiO-66-NH_2 to different functionalized MOFs was determined by comparing the relative integrated areas of the aromatic resonances (corresponding to the C-3 position of the BDC ring) between the modified and unmodified BDC ligands.[1]

1. S. J. Garibay and S. M. Cohen, *Chem. Commun.*, 2010, **46**, 7700-7702.

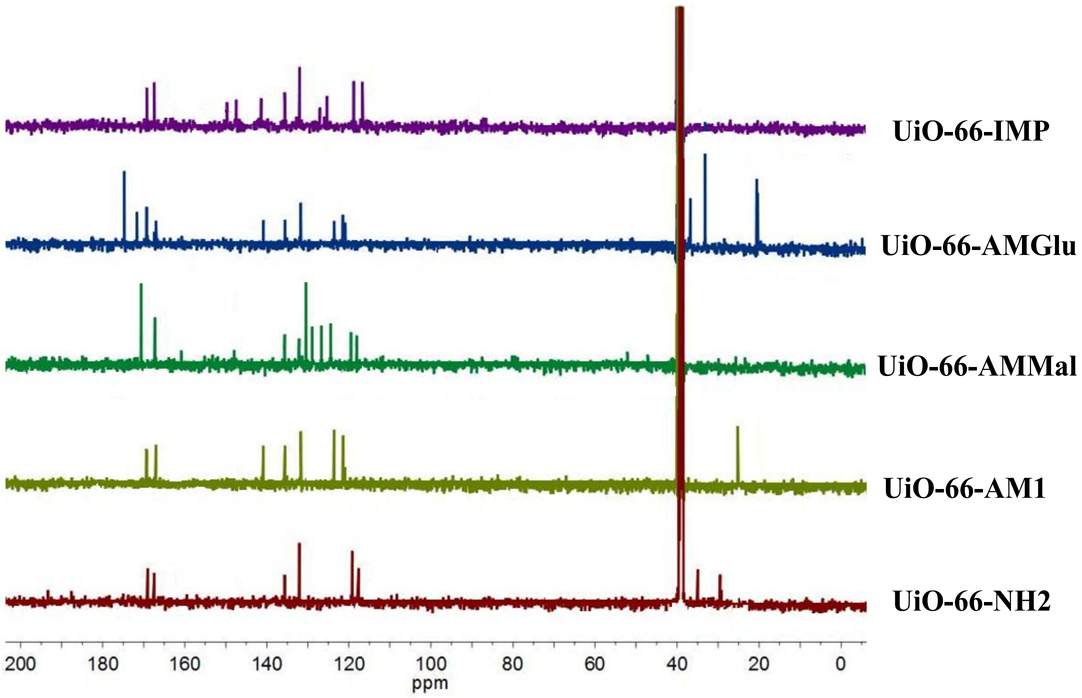

Figure S1. 500 MHz ^{13}C-NMR spectra (DMSO-d_6) of UiO-66-NH_2 and different functionalized MOFs

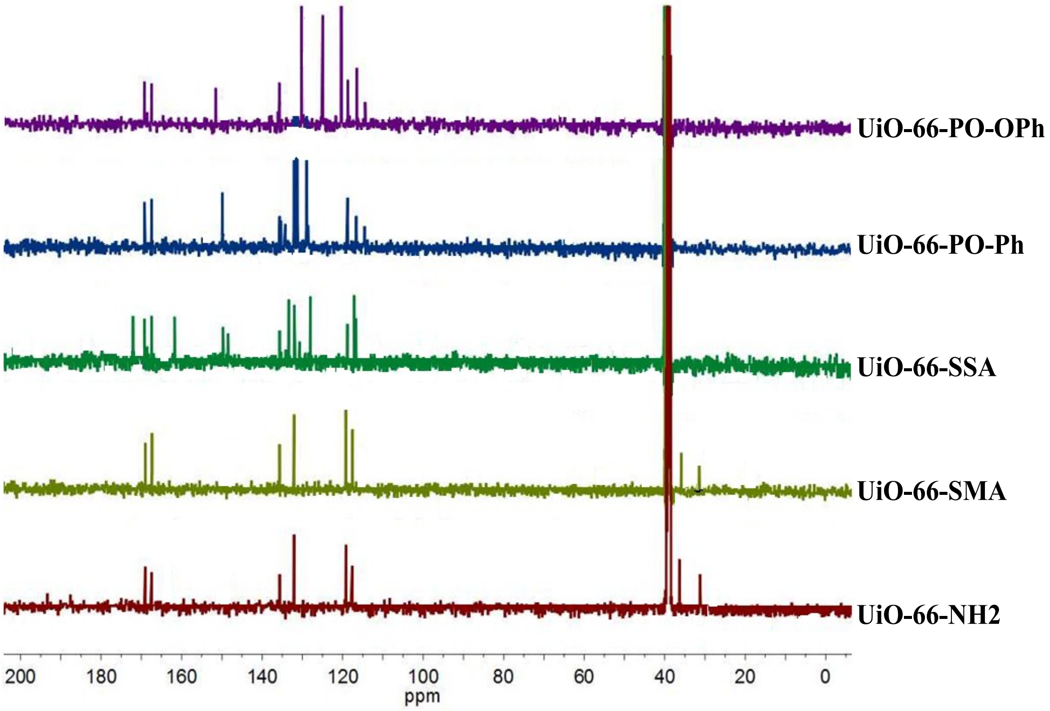

Figure S2. 500 MHz ^{13}C-NMR spectra (DMSO-d_6) of UiO-66-NH_2 and different functionalized MOFs

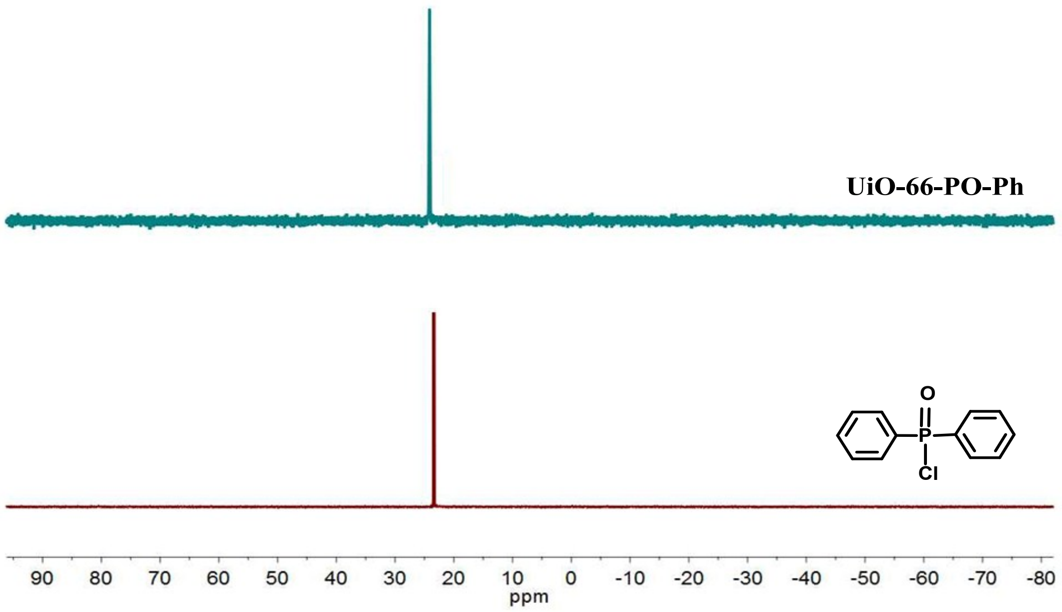

Figure S3. ^{31}P NMR (600 MHz, ^{1}H decoupled) in DMSO-d_6.

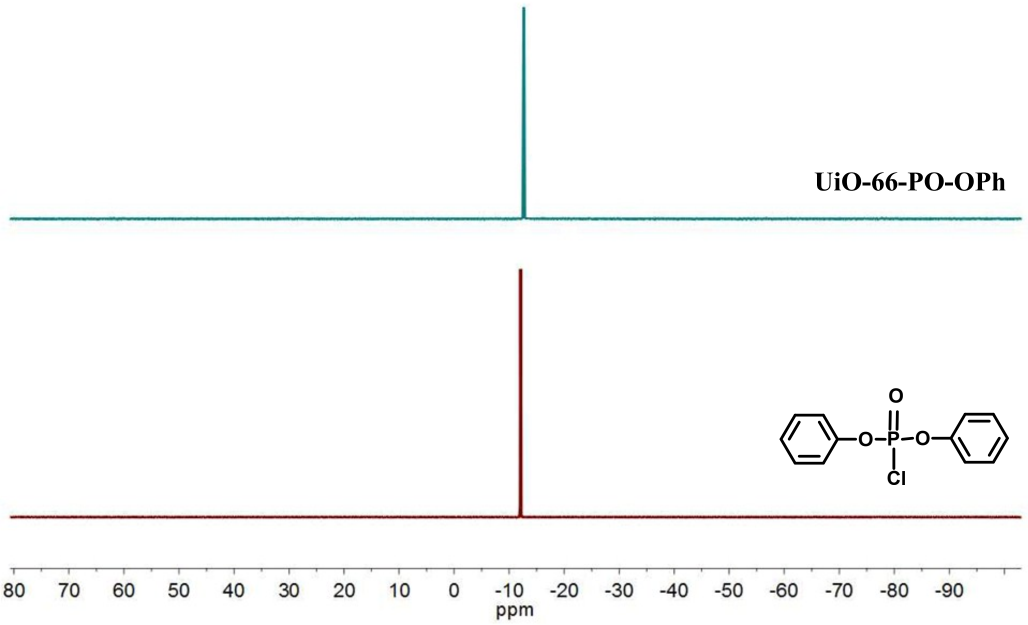

Figure S4. ^{31}P NMR (600 MHz, ^{1}H decoupled) in DMSO-d_6.

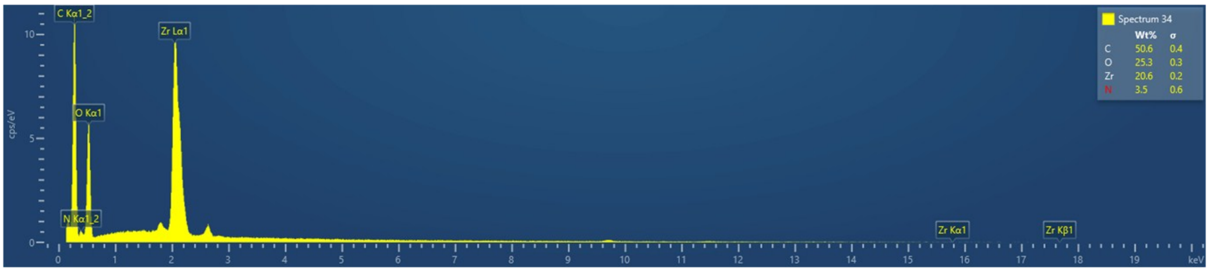

(a)

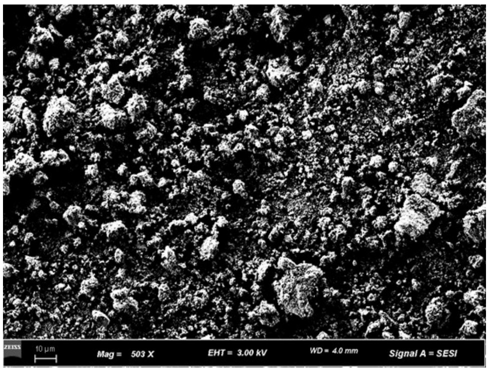

(b)

Figure S5. (a) SEM and (b) EDX spectra of UiO-66-NH_2.

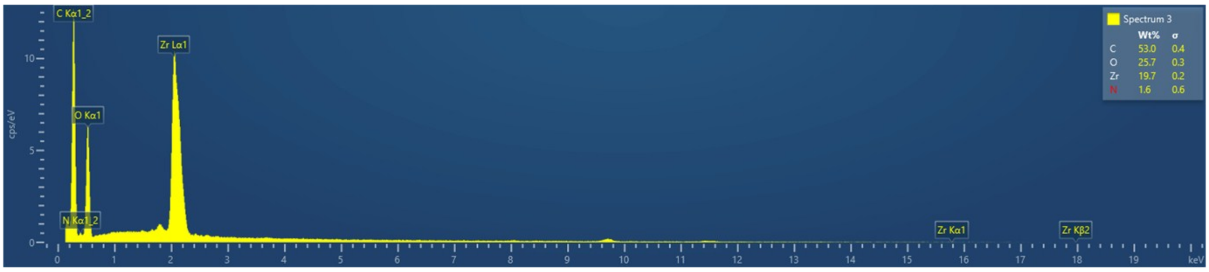

(a)

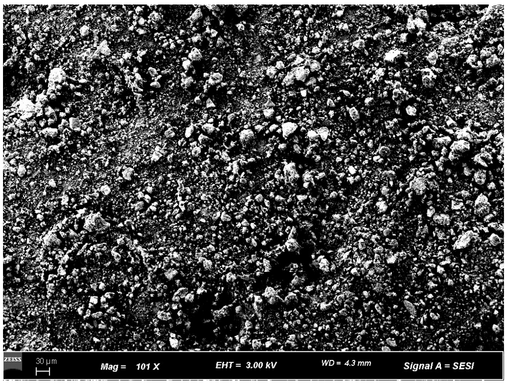

(b)

Figure S6. (a) SEM and (b) EDX spectra of UiO-66-AM1.

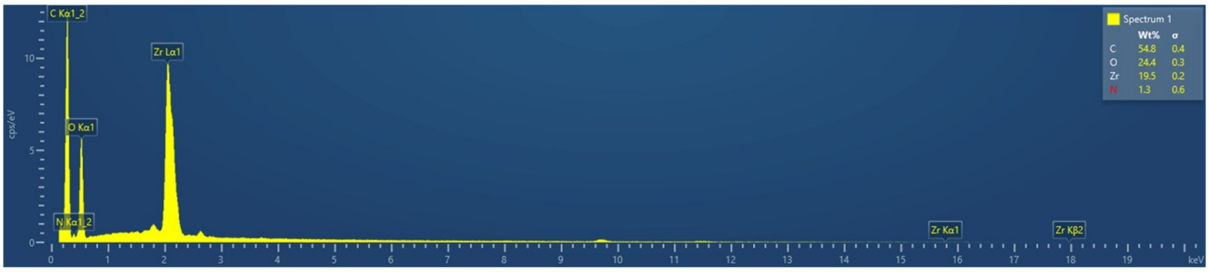

(a)

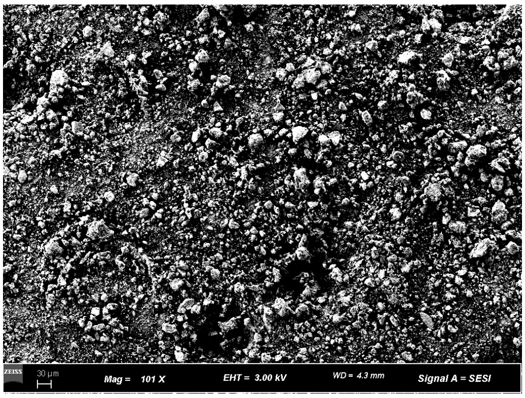

(b)

Figure S7. (a) SEM and (b) EDX spectra of UiO-66-AMMal.

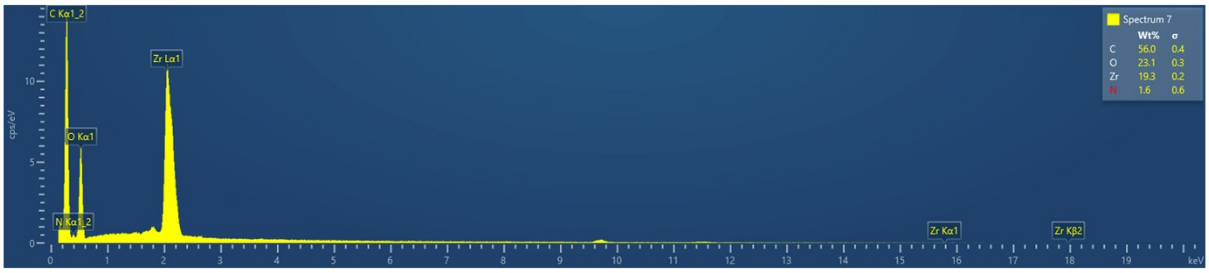

(a)

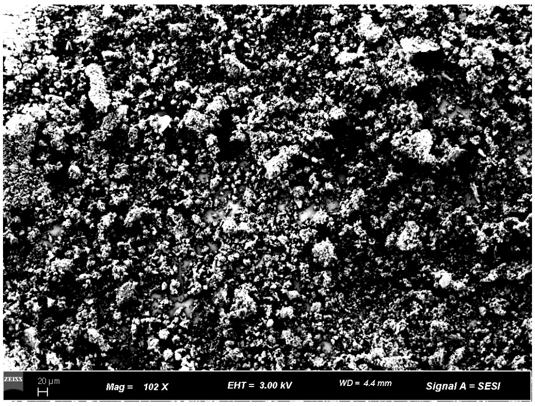

(b)

Figure S8. (a) SEM and (b) EDX spectra of UiO-66-AMGlu.

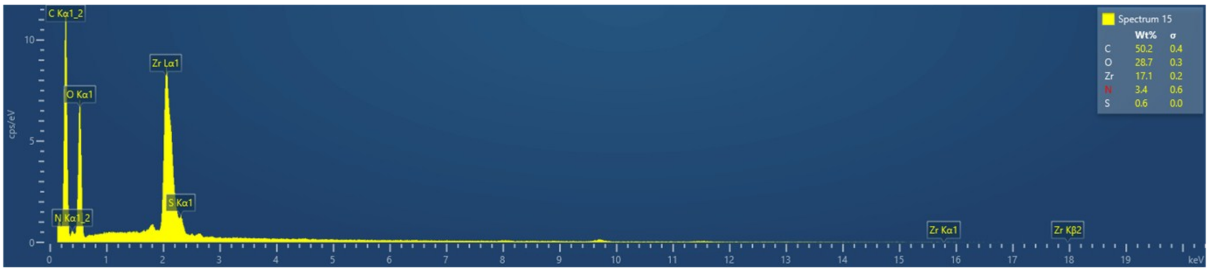

(a)

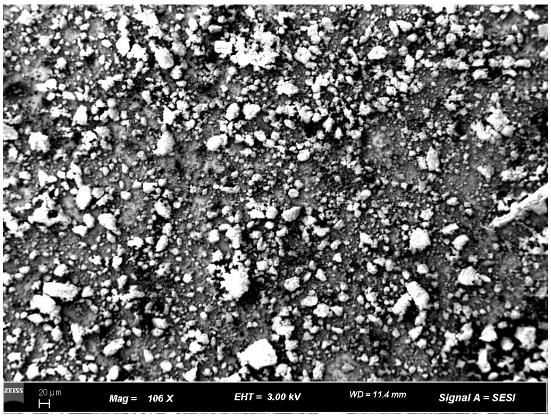

(b)

Figure S9. (a) SEM and (b) EDX spectra of UiO-66-SMA.

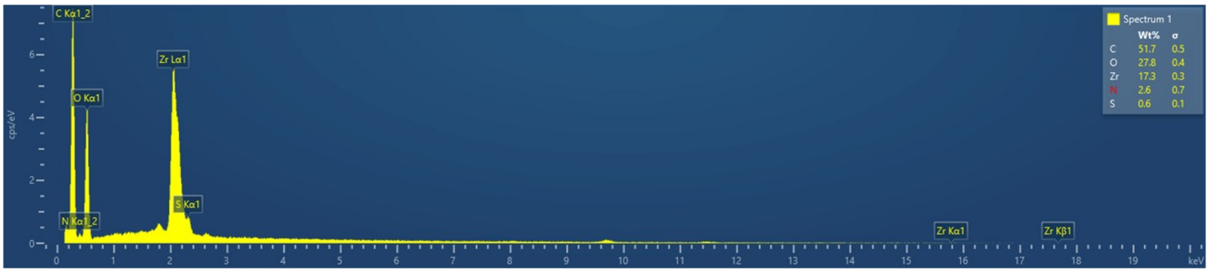

(a)

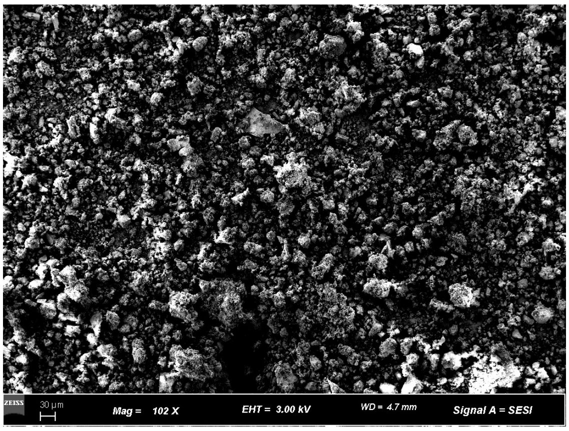

(b)

Figure S10. (a) SEM and (b) EDX spectra of UiO-66-SSA.

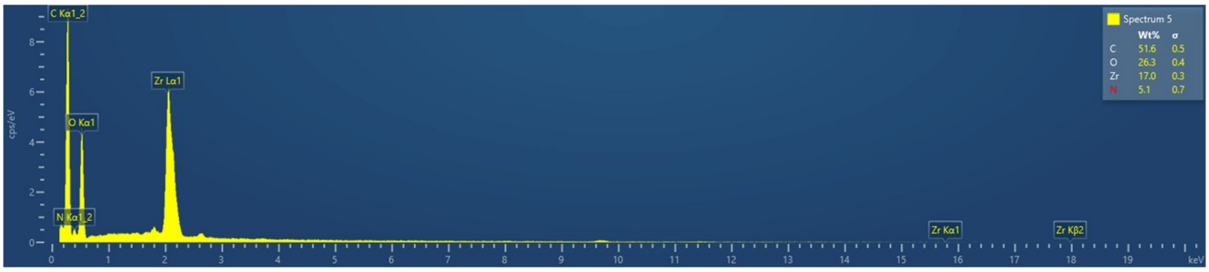

(a)

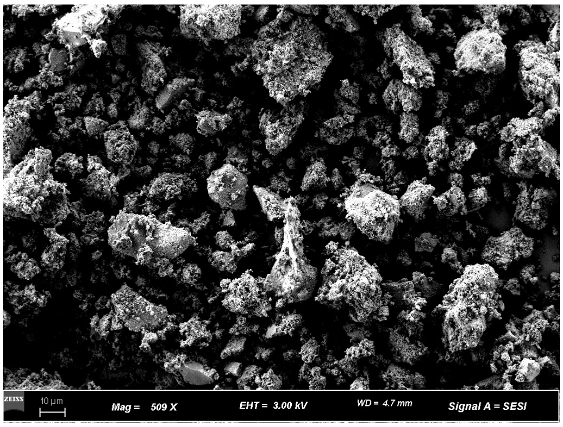

(b)

Figure S11. (a) SEM and (b) EDX spectra of UiO-66-IMP.

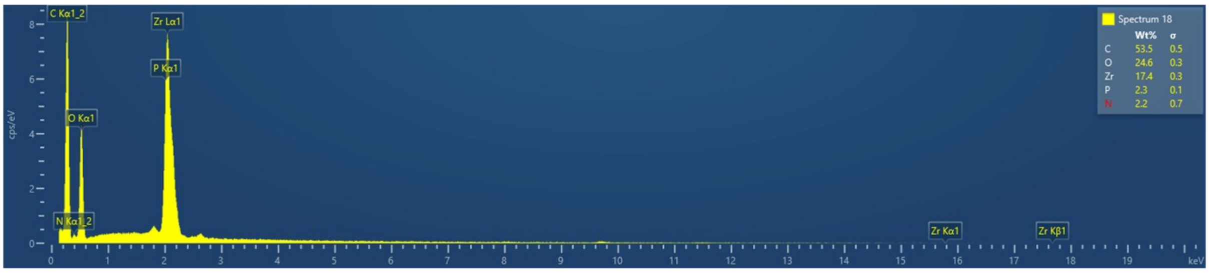

(a)

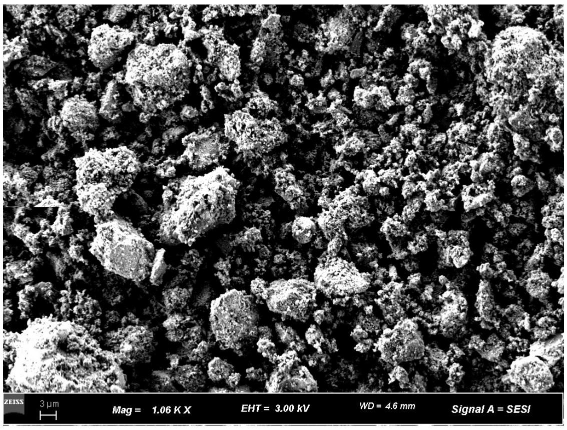

(b)

Figure S12. (a) SEM and (b) EDX spectra of UiO-66-PO-Ph.

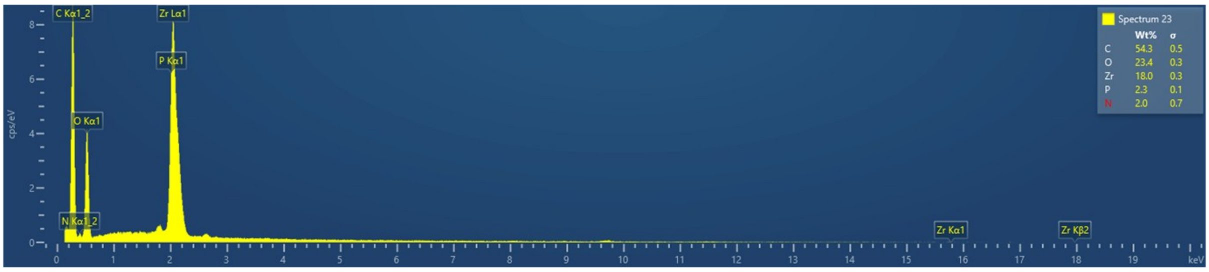

(a)

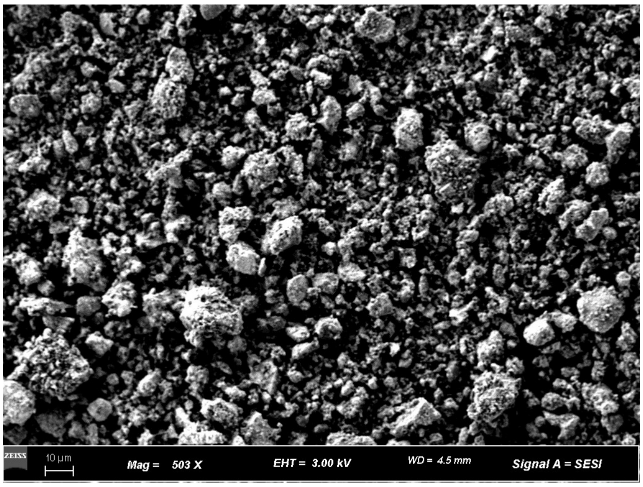

(b)

Figure S13. (a) SEM and (b) EDX spectra of UiO-66-PO-OPh.

Table S2. BET surface area of UiO-66-NH_2 and different functionalized MOFs

MOF	BET Surface area (m^2g^{-1})
UiO-66-NH_2	1112[1]
UiO-66-AM1	818[1]
UiO-66-AMMal	814[1]
UiO-66-AMGlu	531
UiO-66-IMP	653
UiO-66-SMA	748
UiO-66-SSA	595
UiO-66-PO-Ph	609
UiO-66-PO-OPh	574

1. Garibay, S. J.; Cohen, S. M. *Chem. Commun.* **2010**, *46*, 7700-7702.

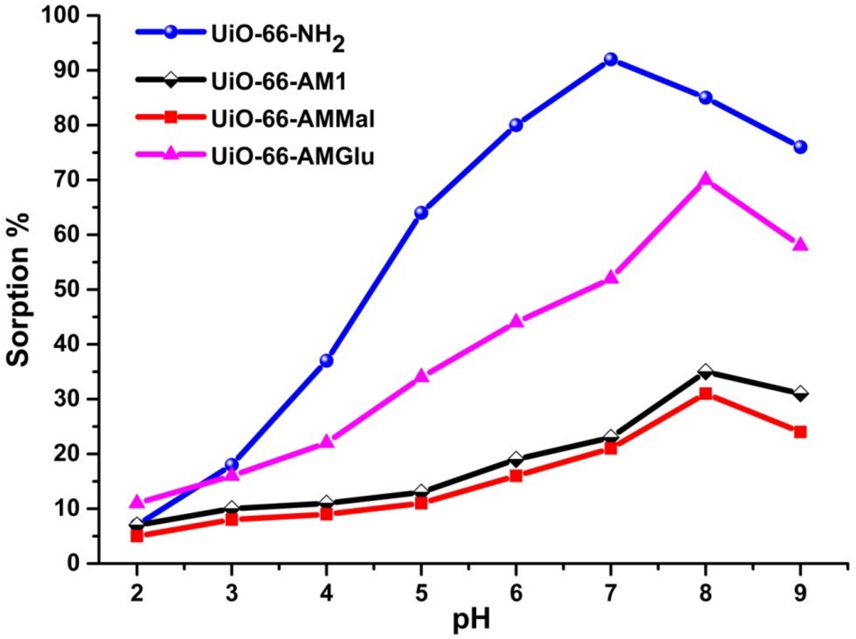

Figure S14. Effect of pH on uranyl ion sorption onto UiO-66-NH_2, UiO-66-AM1, UiO-66-AMMal and UiO-66-AMGlu; t = 120 min, $m_{sorbent}$ = 10.0 mg, $V_{solution}$ = 3 mL, C_0 =500 mg L^{-1}, T = 25±0.5 °C.

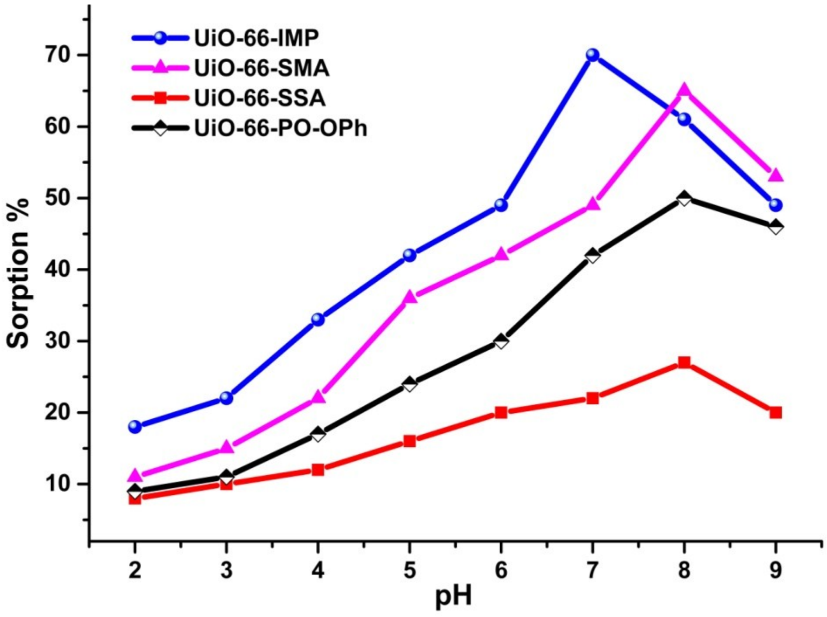

Figure S15. Effect of pH on uranyl ion sorption onto UiO-66-IMP, UiO-66-SMA, UiO-66-SSA and UiO-66-PO-OPh; t = 120 min, $m_{sorbent}$ = 10.0 mg, $V_{solution}$ = 3 mL, C_0 =500 mg L^{-1}, T = 25±0.5 °C.

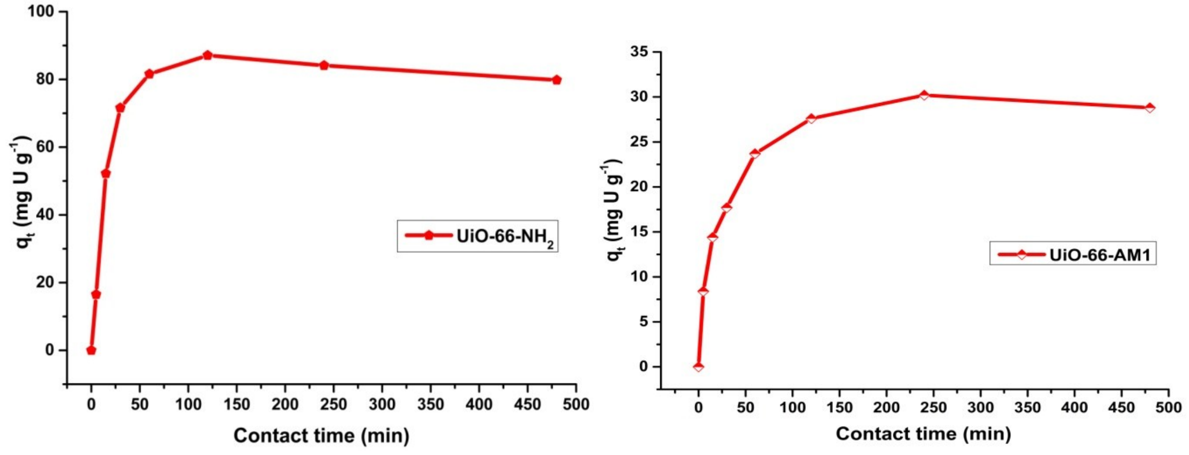

(a) (b)

Figure S16. Effect of contact time on uranyl ion sorption onto (a) UiO-66-NH$_2$ (pH = 7), (b) UiO-66-AM1 (pH = 8); $m_{sorbent}$ = 10.0 mg; $V_{solution}$ = 3 mL; C_0 =500 mg L^{-1}; T = 25±0.5 °C.

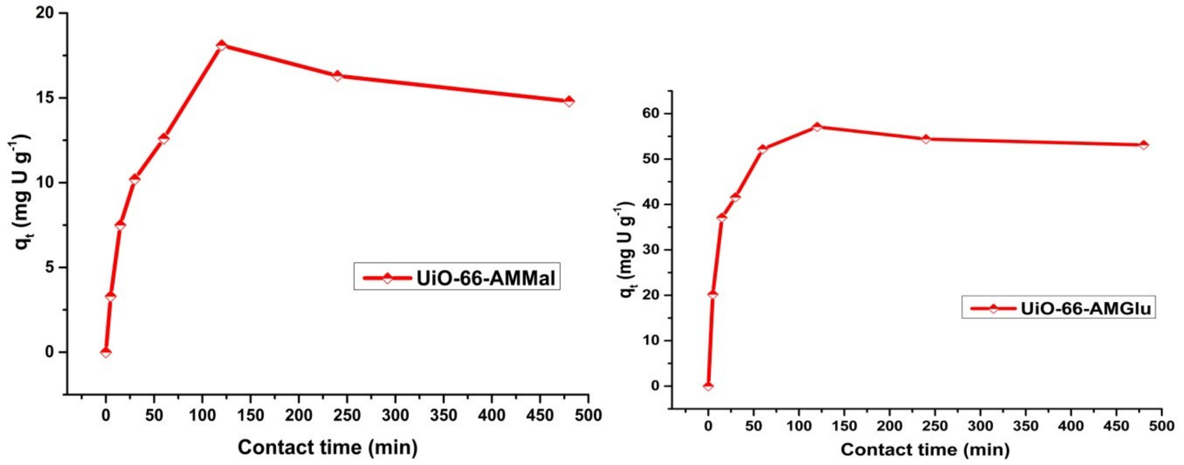

(a) (b)

Figure S17. Effect of contact time on uranyl ion sorption onto (a) UiO-66-AMMal (pH = 8), (b) UiO-66-AMGlu (pH = 8); $m_{sorbent}$ = 10.0 mg; $V_{solution}$ = 3 mL; C_0 =500 mg L^{-1}; T = 25±0.5 °C.

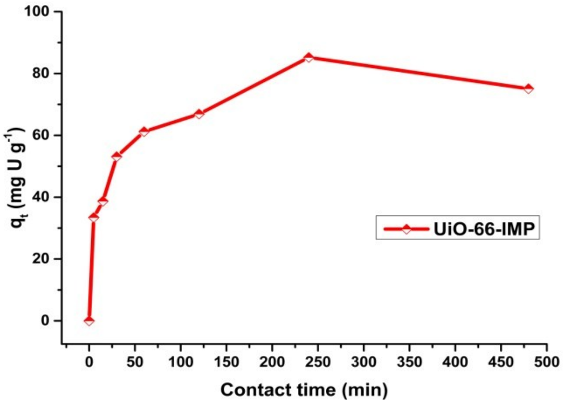

(a)

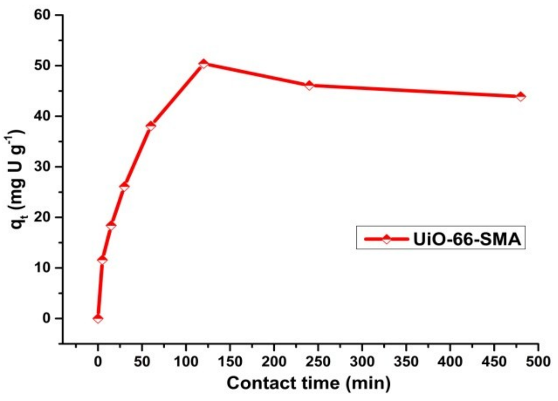

(b)

Figure S18. Effect of contact time on uranyl ion sorption onto (a) UiO-66-IMP (pH = 7), (b) UiO-66-SMA (pH = 8); m_{sorbent} = 10.0 mg; V_{solution} = 3 mL; C_0 =500 mg L^{-1}; T = 25±0.5 °C.

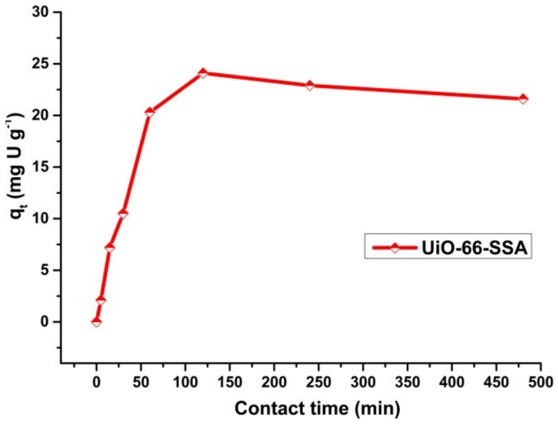

(a)

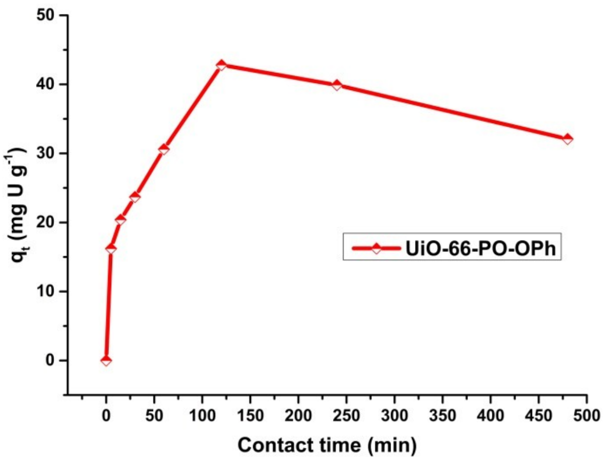

(b)

Figure S19. Effect of contact time on uranyl ion sorption onto (a) UiO-66-SSA (pH = 8), (b) UiO-66-PO-OPh (pH = 8); m_{sorbent} = 10.0 mg; V_{solution} = 3 mL; C_0 =500 mg L^{-1}; T = 25±0.5 °C.

Kinetic model

The linear form of the pseudo-second-order kinetic model is expressed as follows:

$$\frac{t}{q_t} = \frac{1}{K_2 q_t^2} + \frac{t}{q_e}$$

Where q_e (mg·g^{-1}) and q_t (mg·g^{-1}) are the amounts of the uranium absorption at equilibrium and at time t, respectively. And K_2 (g·mg^{-1}·min^{-1}) is the pseudo-second-order sorption rate constant. The model parameters and the correlation coefficient obtained are shown in Table S2.

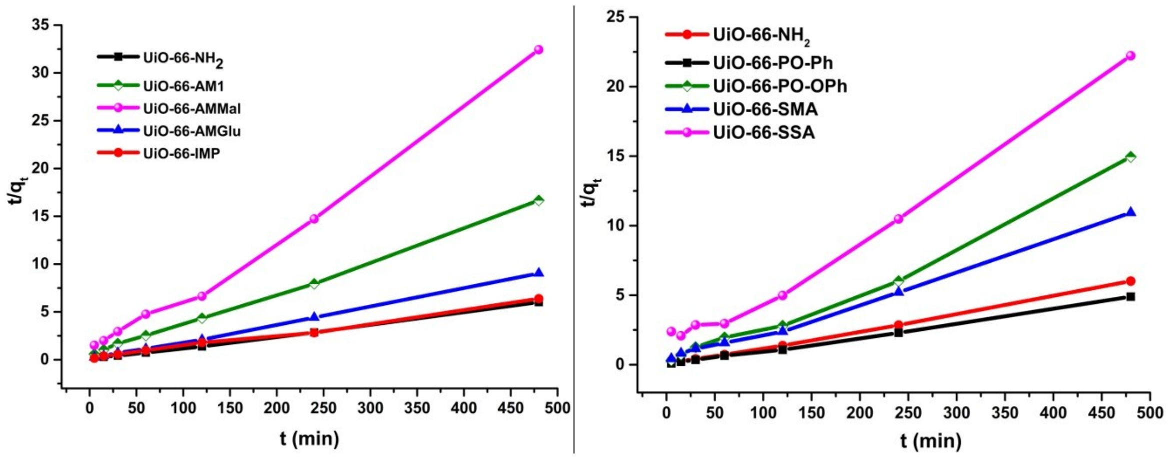

Figure S20. Pseudo-second-order model fits for the kinetic rate on the sorbent.

Table S3. Kinetic parameters for uranyl ion sorption on the MOF

Pseudo-second-order kinetic model			
**MOF**	q_e (mg·g^{-1})	K_2 (g·mg^{-1}·min^{-1})	R^2
UiO-66-PO-Ph	105.4	0.0029	0.996
UiO-66-NH_2	82.1	0.0058	0.991
UiO-66-IMP	78.3	0.0014	0.992
UiO-66-AMGlu	53.0	0.00185	0.998
UiO-66-SMA	46.4	0.00217	0.993
UiO-66-PO-OPh	36.1	0.0029	0.981
UiO-66-AM1	27.2	0.0033	0.997
UiO-66-AMMal	23.1	0.0064	0.989
UiO-66-SSA	21.4	0.0042	0.982

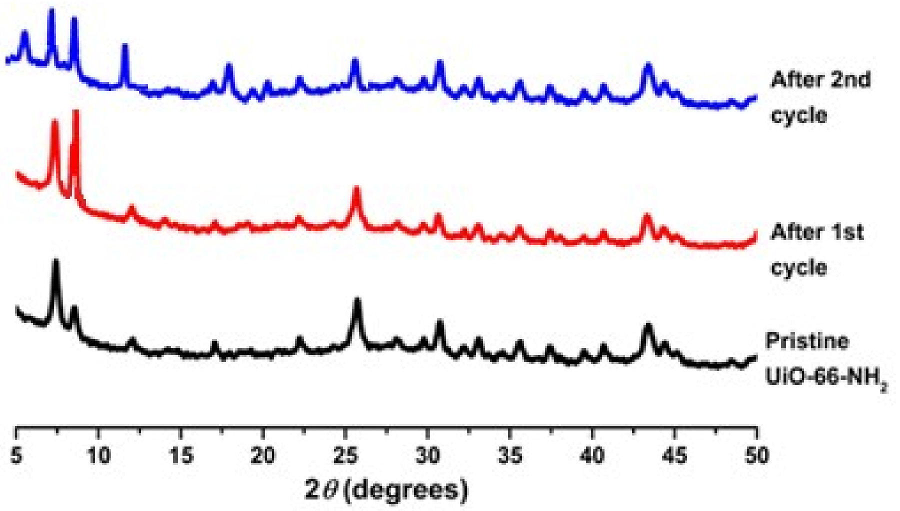

Figure S21. Powder XRD pattern of pristine UiO-66-NH_2 and after two cycles of uranyl ion sorption.

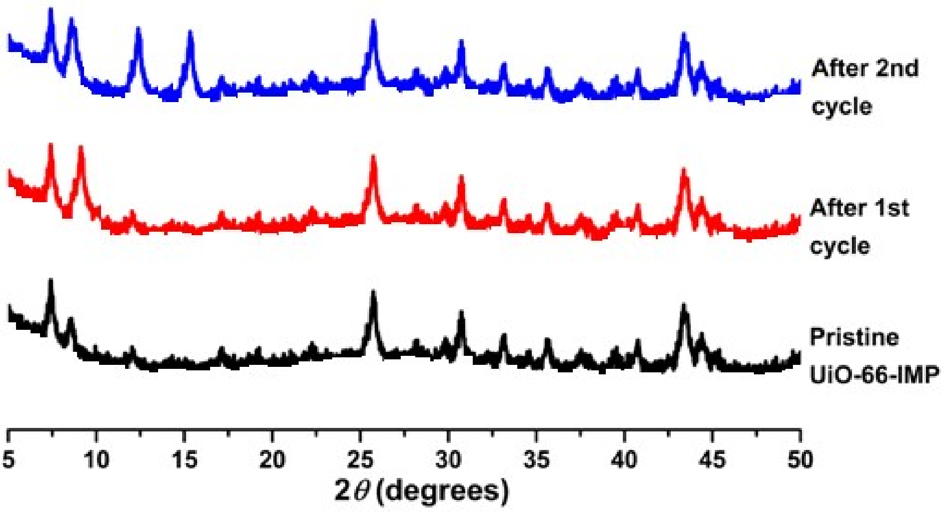

Figure S22. Powder XRD pattern of pristine UiO-66-IMP and after two cycles of uranyl ion sorption.

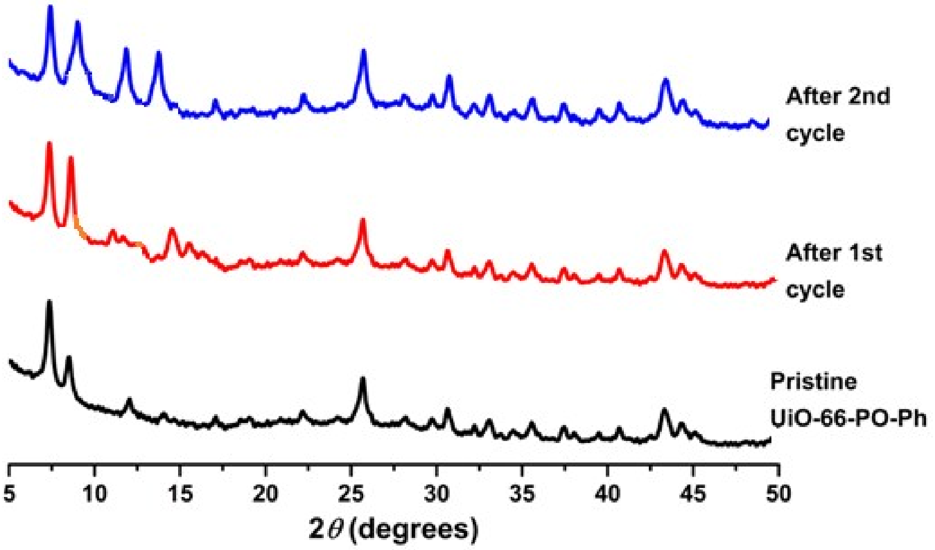

Figure S23. Powder XRD pattern of pristine UiO-66-PO-Ph and after two cycles of uranyl ion sorption.
